# Dissecting the genetic basis of relevant fruit quality traits in interspecific grapevines (*Vitis* spp.)

**DOI:** 10.1093/hr/uhaf353

**Published:** 2025-01-06

**Authors:** Venkateswara Rao Kadium, Ramesh Pilli, Andrej Svyantek, Zhuoyu Wang, John Stenger, Rajasekharreddy Bhoomireddy, Collin Auwarter, Xuehui Li, Harlene Hatterman-Valenti

**Affiliations:** Department of Plant Sciences, North Dakota State University, Fargo, ND 58102, USA; Department of Plant Sciences and Plant Pathology, Montana State University, Bozeman, MT 59717, USA; Department of Horticulture Sciences, Texas A&M University, College Station, TX 77843, USA; Department of Plant Sciences, North Dakota State University, Fargo, ND 58102, USA; Department of Plant Sciences, North Dakota State University, Fargo, ND 58102, USA; Department of Horticulture Sciences, Texas A&M University, College Station, TX 77843, USA; Department of Plant Sciences, North Dakota State University, Fargo, ND 58102, USA; Department of Food Science and Technology, Texas A&M University, College Station, TX 77843, USA; School of Natural Resource Sciences, North Dakota State University, Fargo, ND 58102, USA; Department of Plant Sciences, North Dakota State University, Fargo, ND 58102, USA; Department of Plant Sciences, North Dakota State University, Fargo, ND 58102, USA; Department of Plant Sciences, North Dakota State University, Fargo, ND 58102, USA; Department of Plant Sciences, North Dakota State University, Fargo, ND 58102, USA

## Abstract

Understanding the genetic control of fruit composition traits in interspecific grapevines (*Vitis* spp.) is crucial when breeding new cultivars with desirable fruit chemistry. To address this, a genome-wide association study (GWAS) was conducted using 587 genotypes derived from three elite selections. This study spanned 3 years (2020–2022) and with phenotyping conducted at three different timepoints within each season for a total of nine phenotyping events focused on nine fruit traits. Several strong and stable quantitative trait locus (QTL) associations were identified on chromosomes 6, 16, and 17 across multiple phenotyping events for most sugar- and acid-related traits. Notably, putative sugar transporter genes Vitvi16g00860 and Vitvi16g00861 on chromosome 16, which facilitate the movement of sugars and K+ ions across membranes, were found to be associated with all sugar and acid traits studied. Additionally, several QTLs on chromosomes 1–5, 7, 14, and 18 were identified for various fruit quality traits across different phenotyping events. We determined functional connections between traits and scrutinized candidate genes by utilizing gene ontology annotations for genes located near significant SNPs. We also highlighted the effect of different forms of phenotype (best linear unbiased predictions and unmodified) in suppressing certain QTL associations. This GWAS study focused on fruit quality in grapes, establishing a necessary knowledge base regarding the genetic architecture of these traits to aid molecular breeders in further improving them.

## Introduction

Grapevines (*Vitis* spp.) are among the most significant global horticultural crops with production of >61 million tons of fruit annually; more than half of the total crop is dedicated to wine production [1]. Numerous wine attributes and styles are dictated by the types of organic acids and sugars and their relative concentration within grape berries. The dominant organic acid compounds present in grape berries are malic and tartaric acid, which constitute ~90% of total acids. High levels of malic acid led to an overly sour taste, negatively impacting fruit, juice, and wine. By contrast, high tartaric acid levels are less problematic due to its limited solubility in wine [2]. Tartaric acid accumulates early in berry development and remains constant after veraison. Decrease in tartaric acid concentration is mainly due to dilution because of increases in berry volume during ripening [3]. Malic acid accumulates and reaches its maximum concentration just prior to veraison [4]. Malic acid is involved in several essential metabolic pathways where it is used as a carbon energy source [5]. Malate oxidation along with sugar accumulation during ripening leads to reduction in total acid content that drives alterations in acid–sugar balance of the berries. Another important parameter of fruit and wine is pH. Maintaining low pH of wines is essential to prevent microbial spoilage [5]. There is an inverse correlation between pH and total acids, which is affected by differences in organic acid compositions and the partial exchange of titratable protons, particularly potassium (K^+^) [6]. At maturity the predominant sugars present in berries are glucose and fructose [7]. In terms of perceived sweetness fructose is ~2.33 times sweeter than glucose. The amount of alcohol in wine is dictated by the sugar concentration of berries because yeast converts sugars into alcohol during fermentation.

Organic acids and sugars are complex quantitative traits and their composition in grapes is influenced by cultivar, environment, management, and the interplay among these factors known as genotype × environment × management (G × E × M) interactions. Previous research indicates that grapes grown in cooler areas generally have higher malic acid concentrations, whereas those grown in warmer regions tend to exhibit lower acidity. This inverse relationship between temperature and malic acid levels is attributed to temperature’s impact on malate dehydrogenase activity [8]. In several parts of the USA, the cultivation of *Vitis vinifera* is significantly hindered by various abiotic and biotic stresses, including cold hardiness, frost damage, diseases, and pest infestations, for which *V. vinifera* cultivars lack tolerance or resistance mechanisms [9]. Consequently, viticulture in these regions is dominated by hybrids derived from a combination of American *Vitis* species and *V. vinifera* cultivars. These American *Vitis* species contribute essential tolerance and resistance loci to combat abiotic and biotic stresses. However, in terms of fruit composition, non-*V. Vinfera* species often deviate from the chemical parameters established with *V. vinifera*. In general, these hybrid grapevines present several challenges for wine production due to their high organic acid, low pH, and variable sugar concentration due to poor ripening from limited growing degree day accumulation, short length of season, and varying genetic backgrounds [10, 11]. Given the importance of achieving the ideal sugar-to-acid balance in berries for wine production with minimal amelioration, there is a need to continue breeding new hybrid varieties suitable to these regions.

Traditional grapevine breeding methods encounter difficulties in achieving a balance among fruit quality traits because of the significant time and resources required for thorough phenotyping and selection. This challenge is exacerbated by the extended juvenile phase typical of woody perennial species [12]. In the case of grapes, this is especially relevant as the evaluation of fruit quality traits can only begin once the plant reaches maturity, which require as many as ≥4 years [13]. By traditional breeding techniques it takes ~25–30 years to release a new variety of grapevine. Marker-assisted selection (MAS) using markers linked to trait of interest leads to shortening of breeding cycle in grapevines [14]. MAS can be used to eliminate inferior seedlings, which can help in resource saving by cutting cost up to 16%–34% [15].

Previously, several studies assessed the genetic foundation of sugar and acid traits in grapevines via quantitative trait locus (QTL) mapping using biparental populations. Potential QTLs were identified for total soluble solids (TSS) [4, 16], titratable acidity (TA) [17], glucose [4, 18], fructose [4, 18], tartaric acid [4, 19], malic acid [4, 16, 19], and pH [19, 20]. Many of the QTLs identified varied greatly across populations. This is due to inherent drawbacks of QTL analysis owing to limited allelic diversity from parents and resulting small population size. Biparental mapping accounts for only a few recent recombination events, and it is an inefficient technique for estimation of polygenic control of highly quantitative traits [21]. Genome-wide association studies (GWAS) provide an alternative mapping strategy to this problem by accounting for functional variation in a broader context based on linkage disequilibrium (LD) [22]. The genetic diversity present in the genotypes of the association panel stems from numerous historical and evolutionary recombination events occurring over past generations leading to enhanced mapping resolution [23]. GWAS is particularly valuable for species with lengthy generation cycles, such as grapevines, because it eliminates the need for creating mapping populations; however, it can also be used to leverage the genetic variation of multiple populations simultaneously. Previously several studies have used multiparental populations to perform GWAS to identify markers linked to trait of interest [24, 25]. Various association studies have been conducted on grapevines, primarily focusing on traits such as flower sex, cluster size-related traits, berry shape-related traits, berry weight, skin color, muscat aroma, and several postharvest traits [13, 26–30]. Previously, Flure *et al*. conducted GWAS on multiple fruit-related traits using 279 *V. vinifera* L. cultivars. This study, however, employs hybrid genotypes derived from multiple *Vitis* species to perform GWAS on multiple fruit quality traits.

To improve the understanding of genetic architecture behind nine different fruit quality traits (TSS, pH, TA, glucose, fructose, malic, tartaric, citric, and berry mass), an association study was performed at three different time points after veraison using a population of 587 genotypes derived from three elite selections of NDSU grape germplasm enhancement program (GGEP). This population has background from *V. vinifera*, *Vitis riparia*, *Vitis labrusca*, and multiple other *V. spp.* that provide cold hardiness characteristics critical for vine survival in colder region along with needed diversity for fruit quality traits required for GWAS analysis. Major objectives of this study were (i) to discover novel QTLs controlling the major fruit quality traits, (ii) to validate QTLs found in previous studies, and (iii) to perform gene ontology (GO) studies of genes around the significant Single Nucleotide Polymorphisms (SNPs) to elucidate the functional relation between traits.

## Results

### Phenotypic data

**Figure 1:**
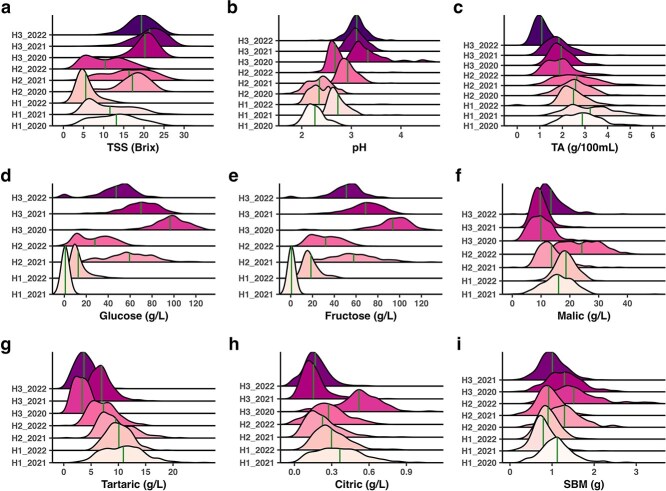
Frequency distribution (density ridge plots) of fruit quality traits (a) TSS, (b) pH, (c) TA, (d) glucose, (e) fructose, (f) malic acid, (g) tartaric acid, (h) citric acid, and (i) SBM by year and harvest. The green vertical line depicts mean value of each distribution. Here H1, H2, and H3 indicate harvest one, two, and three, respectively.

Figure 1 represents the phenotype distribution of nine fruit quality traits measured in the population across 3 years and harvests. Sugar-related traits (TSS, Glucose, and Fructose), pH, and single-berry mass (SBM) displayed a gradual increase in trait value from H1 to H3, whereas acid traits (TA, malic, tartaric, and citric) showed a decreasing trend from H1 to H3 in all 3 years (Fig. 1). The average phenotypic value across harvests and years for traits were as follows: TSS: 14.53°Brix, pH: 2.77, TA: 2.30 g/100 ml, glucose: 37.93 g/l, fructose: 39.35 g/l, malic acid: 15.70 g/l, tartaric acid: 7.87 g/l, citric acid: 0.27 g/l, and SBM: 1.07 g. More detailed summary statistics of each trait are presented in Supplementary Table S1.

### Population structure and linkage disequilibrium

**Figure 2:**
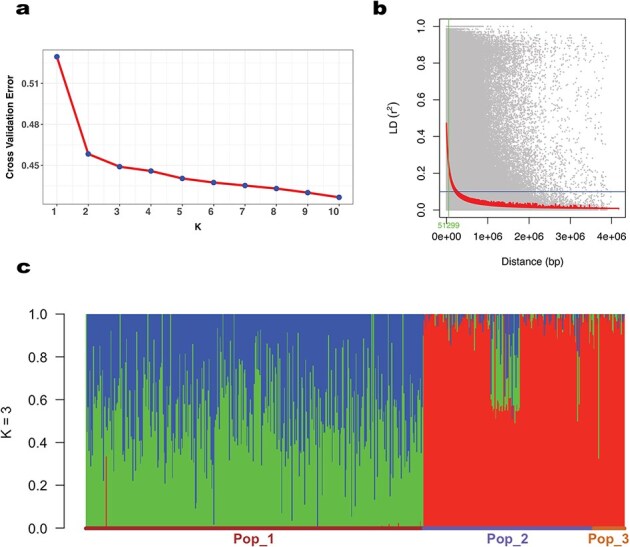
Population structure and LD based on marker data of the population. (a) *K*-value cross-validation plot. (b) LD decay plot of the population (c) ADMIXTURE bar plot for *K* = 1 to 10, where each genotype is represented by a vertical line and each color represents population proportion.

Population structure of 587 individuals was analyzed with the help of ADMIXTURE software. It used population SNP data to calculate maximum likelihood estimation of individual ancestries. Initially the model assumed that the genotype data are a mixture of contributions from *K* = 1–10 ancestral populations. The algorithm iteratively estimated the allele frequencies in each of the *K* (1–10) populations and the proportion of ancestry each individual derives from each population. The optimal value of *K* was estimated based on decline in cross-validation error rate. Cross-validation error reduced from *K* = 1 to 3 significantly (Fig. 2a). From *K* = 3 to 10, the decrease in CV error becomes minimal. This suggests that additional populations beyond *K* = 3 provide only a marginal improvement in model accuracy. The Evanno Δ*K* analysis, which measures the rate of change in log-likelihood between successive *K* values, indicated a clear peak at *K* = 3 (Supplementary Fig. S1). Hence the value of *K* = 3 was chosen to best describe the genetic architecture of the population (Fig. 2c). This was also verified using PCA analysis (Supplementary Fig. S2). LD analysis was performed in Tassel 5.2 software. A total of 19 518 SNPs were used for pairwise analysis. Nonlinear regression model was used to estimate LD decay with distance in base pair. At *r*^2^ threshold of 0.1, the maximum LD was recorded as 0.47. LD decay of 50% was recorded at a distance of 51.29 kb (Fig. 2b).

### Genome-wide association analysis

**Figure 3:**
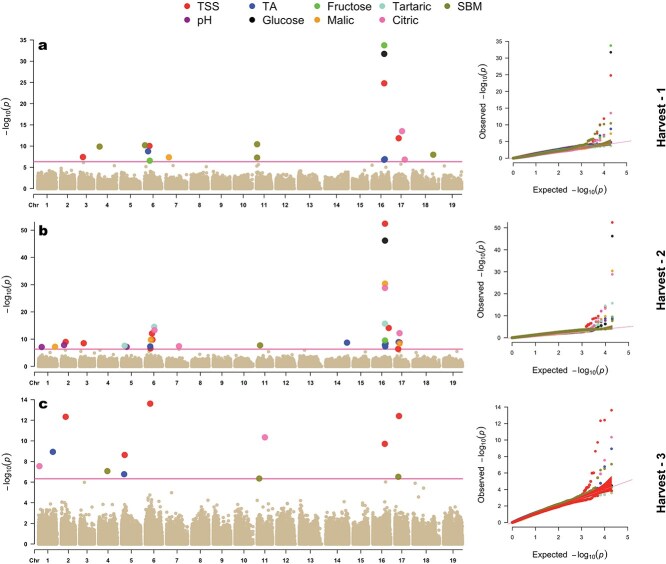
Multitrait Manhattan plots along with their Q-Q plots during (a) Harvest-1, (b) Harvest-2, and (c) Harvest-3 for nine fruit quality traits studied. The pink vertical line denotes significant threshold at Bonferroni correction of 0.01 [−log_10_(p) = 6.32].

Association analysis of nine fruit quality traits (Best Linear Unbiased Predictions (BLUPs)) from 482 fruit genotypes with 19 518 SNP markers was performed using Blink algorithm in GAPIT3 package to detect marker–trait associations. A deviation from observed *P*-value to expected *P*-value was observed in Q-Q plots for all nine traits studied across different harvest events. Only the associations that surpassed stringent Bonferroni threshold at 0.01 level (−log_10_P = 6.32) were considered significant. Association analysis using the anthocyanin trait also identified a previously reported, well-established QTL on chr 2 [31, 32], indicating good model fit within the studied population (Supplementary Fig. S3). Significant hits during three harvest time points (H1–H3) were slightly varied among traits in regard to the number of hits and chromosome location of specific marker–trait associations. During H2 (32 marker–trait associations for nine different traits) the highest number of traits showed significant associations; this was followed by H1 (18 marker–trait associations for seven different traits) and H3 (13 marker–trait associations for four different traits) (Fig. 3 and Table 1). The majority of trait associations were concentrated on three chromosomes. Specifically, chr 16 had the highest number of GWAS hits followed by chr 6, and chr 17 with 16, 12, and 10 association on each, respectively. Along with them, chr 1–5, 7, 11, 14, and 18 also had marker–trait associations detected. Among the nine traits with significant hits, glucose had the fewest associations (2), while TSS (18), SBM (10), TA (10), and citric acid (8) had the highest number of associations (Fig. 3). SNP chr16_15992931 was associated with four traits (fructose, glucose, malic acid, and tartaric acid), whereas SNPs chr16_15991560 (1.3 kb away from the earlier mentioned SNP, chr16_15992931), chr6_6497421, and chr17_6001448 were associated with two traits each (Table 1). Traditional QTL analysis conducted for trait TSS also identified significant QTLs on chromosomes 6, 16, and 17 in multiple harvests. (Supplementary Fig. S4).

### Candidate gene and gene ontologies

**Figure 4:**
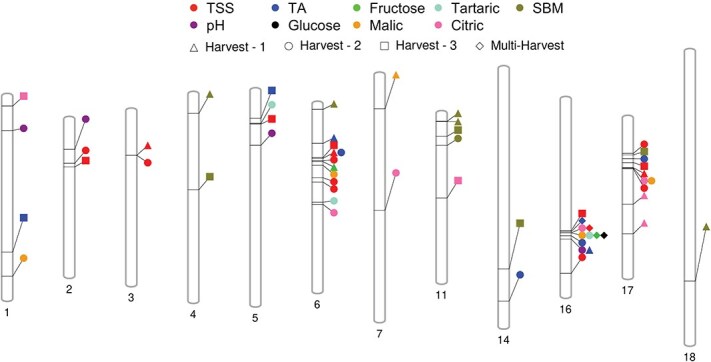
Phenogram showing chromosomal localization of 52 significant SNPs identified for fruit quality traits in the GWAS analysis.

Blink algorithm determined 52 distinct SNPs associated with nine fruit traits in the assessed population during three harvests (Fig. 4). A total of 31 of these SNPs were found to be located within a gene, while 19 SNPs were located within 6 kb distance from a gene. The nearest genes for the remaining two SNPs (TA_H1 – chr16_15872778, TSS_H2 – chr17_4241609) were located at 10.87 and 16.57 kb, respectively. There were a total of 139 distinct genes within a 20-kb region around significant SNPs (Supplementary Table S2). On average, each GWAS hit was associated with 2.64 genes. Four of these genes were shared between multiple hits, whereas the remaining 136 genes were associated with only one distinct SNP. The number of genes found during each harvest were 44, 68, and 38 for H1, H2, and H3, respectively, with 10 identified genes shared between multiple harvests. Genes Vitvi16g00860 and Vitvi16g00861 were common for all sugar (TSS, glucose, and fructose) and acid (malic, tartaric, and citric) traits. We observed only a single gene in the 20-kb region for 10 hits and a maximum of six genes for SNP chr6_6497421.

Out of 139 total candidate genes, 75 (54%) had at least one GO annotation. This resulted in a total of 288 GO terms, with an average of 2 GO terms per gene. Based on function, GOs were classified into three distinct categories: biological process (85 GO terms), cellular component (115 GO terms), and molecular function (88 GO terms). The most frequently annotated GO terms for cellular components (CC) were ‘GO:0016020 membrane’ (22), ‘GO:0005634 nucleus’ (11), and ‘GO:0005737 cytoplasm’ (10). For molecular function (MF): ‘GO:0005524 ATP binding’ (14), ‘GO:0015386 potassium: proton antiporter activity’ (9), and ‘GO:0003700 DNA-binding transcription factor activity’ (5). For biological process (BP): ‘GO:0006486 protein glycosylation’ (5), ‘GO:0016310 phosphorylation’ (5), and ‘GO:0006749 glutathione metabolic process’ (4).

### Correlation between fruit traits

**Figure 5:**
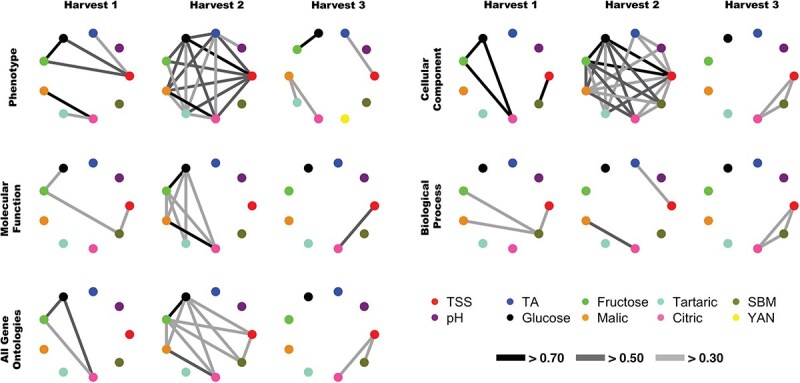
Comparison of network correlations between traits based on GO category and phenotype during different harvests. Strength of connecting line indicates the strength of correlation.

**Figure 6:**
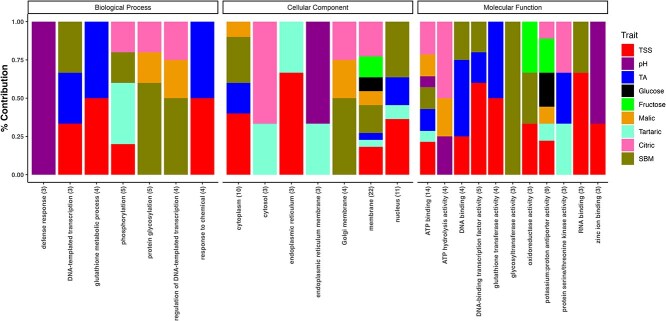
Relative representation of fruit quality traits in contributing to various GO terms that appeared more than twice in the study.

Correlations among trait phenotypes (BLUPs) and GO categories varied depending on harvest time. In general, trait correlations were higher during H2 for both BLUP phenotype and GO variables (Fig. 5). The phenotype of traits glucose, fructose, and TSS were strongly correlated across harvests, this was also true for traits citric, malic, and tartaric. In this study, correlations based on GO variables were generally lower than phenotype-based correlations, except for the CC–GO category, which consists of 13 GO terms that were detected more than once. These strong correlations (*r* > 0.5) in CC–GO were majorly driven by ‘GO:0016020 membrane’, which was observed in all fruit quality traits except pH and ‘GO:0005634 nucleus’, ‘GO:0005737 cytoplasm’, which were observed in traits TSS, TA, Tartaric, and SBM across multiple harvests (Fig. 6).

Correlations based on MF–GO and BP-GO subsets were significantly lower than those observed for phenotypic and CC–GO correlations. In MF–GO, only two correlations were above *r* > 0.7 (between glucose and fructose in H2 and between malic and citric acid during H2) and one correlation was above *r* > 0.5 (between citric acid and TSS during H3). In the BP–GO category, only one correlation was above *r* > 0.5 (the correlation between malic acid and citric acid in H2) (Fig. 5). In the MF–GO subset, correlations (*r* > 0.3) across various harvests were mainly driven by ‘GO:0005524 ATP binding’, which is shared between all traits except glucose and fructose, and ‘GO:0015386 potassium (K^+^) proton antiporter activity’, which is shared between all sugar and acid traits. In the BP–GO subset, correlations (*r* > 0.3) were mainly driven by ‘GO:0016310 phosphorylation’ (Shared between TSS, tartaric acid, citric acid, and SBM), ‘GO:0006486 protein glycosylation’ (Shared between citric acid, malic acid, and SBM) (Fig. 6).

**Table 1:** Continued

**Trait**	**SNP**		−**Log**_**10**_**(p)**	**Nearest gene**	**Dist. (bp)**	**Region annotation**
	H2	chr16_15991560	28.81	Vitvi16g00861	1592	
		chr6_12029388	13.29	Vitvi06g00919	4063	
		chr17_6001448	12.18	Vitvi17g00511	0	Galectin domain-containing protein
		chr7_16205508	7.4	Vitvi07g01172	0	
	H3	chr11_10127081	10.34	Vitvi11g00813	0	Autophagy-related protein 18b
		chr1_1170416	7.55	Vitvi01g00108	0	HMA domain-containing protein
**SBM**	H1	chr11_1004092	10.44	Vitvi11g00093	0	Uncharacterized protein
		chr6_668686	10.2	Vitvi06g00051	0	Tubulin alpha chain
		chr4_2322696	9.88	Vitvi04g00244	0	Dehydrogenase/reductase SDR family member on chromosome X
		chr18_27491643	7.97	Vitvi18g01924	0	Ran-binding protein M-like
		chr11_976113	7.29	Vitvi11g00090	0	12-oxophytodienoate reductase 3 protein (EC 1.3.1.42)
	H2	chr11_3857767	7.71	Vitvi11g00400	0	Sodium/pyruvate cotransporter BASS2, chloroplastic
	H3	chr14_23989210	7.1	Vitvi14g02938	489	Uncharacterized protein
		chr4_11452450	7.07	Vitvi04g00871	5586	UspA domain-containing protein
		chr17_4402253	6.51	Vitvi17g00370	3140	
		chr11_2749482	6.35	Vitvi11g01394	899	Exostosin GT47 domain-containing protein

In the SNP ID, characters before underscore (_) indicates chromosome number and after underscore indicates SNP position; H1, H2, H3 indicates Harvest 1, Harvest 2, and Harvest 3 respectively; Dist.(bp) indicates base pair distance from significant SNP to its nearest gene. More comprehensive information of GWAS results can be found in Supplementary Table S2.

### Role of traits in shaping gene ontologies

Figure 6 demonstrates the most frequent GO terms’ trait contributions. A comparative impact of traits on GO terms depending on the number of significant markers under each trait was observed. However, upon analyzing the GO terms that appeared more than twice, trait specificity was identified. Only six GO terms showed contributions from four or more traits. These are ‘GO:0016020 membrane’ (22 contributions from eight traits), ‘GO:0005524 ATP binding’ (14 contributions from seven traits), ‘GO:0005634 nucleus (11 contributions from four traits)’, ‘GO:0005737 cytoplasm’ (10 contributions from four traits), ‘GO:0015386 potassium (K^+^) proton antiporter activity’ (9 contributions from six traits) and ‘GO:0016310 phosphorylation’ (five contributions from four traits). The three most common GO terms do not have any contribution from trait pH, but ‘defense response’ is exclusively associated with this trait. Likewise, ‘glycosyltransferase activity’ is exclusively associated with SBM.

Regarding sugar traits, TSS had the greatest number of identified SNPs with several genes located within the 20 kb investigated regions. This led to the demarcation of the 16 most common GO terms associated with TSS. Fructose had only two significant hits, of which SNP (chr6_6883931) located within the gene, Vitvi06g00591, associated with ‘GO:0016491 oxidoreductase activity’. Another SNP (chr16_15992931) identified for fructose, glucose, and TSS is located at 2.9 kb distance from the gene Vitvi16g00860. This contributed to GOs ‘GO:0016020 membrane’ and ‘GO:0015386 potassium (K^+^) proton antiporter activity’ shared by all three sugar traits.

Regarding acid-related traits, TA has the highest number of GO terms (11) followed by citric acid (10), tartaric acid (9), and malic acid (8). Four of these GOs were shared by at least three acid traits (Fig. 6). All four acid traits were associated with the GO term ‘GO:0016020 membrane’. The most common acids in grape juice, malic and tartaric, shared three GOs due to the common SNP (chr16_15992931). This SNP on chr 16 is also associated with all sugar traits, as mentioned earlier, indicating the importance of associated GO terms near this SNP in influencing sugar to acid ratio of the berries. There were 10 common GO terms that are shared between traits TA and TSS. Four of these 10 ontologies were shared due to the common SNP chr6_6497421.

**Table 1:** Significant loci associated with fruit quality traits among three different harvests as determined by the Blink algorithm, along with their −log_10_(p) value, nearest gene to SNP, distance from the SNP, and its annotation

**Trait**	**SNP**		−**Log**_**10**_**(p)**	**Nearest gene**	**Dist. (bp)**	**Region annotation**
**TSS**	H1	chr16_15991560	24.8	Vitvi16g00861	1592	
		chr17_5928035	11.85	Vitvi17g00502	0	
		chr6_6497421	10.03	Vitvi06g00556	0	UspA domain-containing protein
		chr3_5316412	7.42	Vitvi03g00492	2009	Autophagy-related protein 2 isoform X2
	H2	chr16_15991560	52.4	Vitvi16g00861	1592	
		chr16_20841143	14.1	Vitvi16g01201	2516	AP2/ERF domain-containing protein
		chr6_8828811	12.08	Vitvi06g00798	0	Myb-like domain-containing protein
		chr6_9365253	9.82	Vitvi06g00841	0	Helicase Helix-turn-helix domain-containing protein
		chr2_5289969	8.97	Vitvi02g01449	5323	
		chr17_6081752	8.76	Vitvi17g00515	0	WW domain-binding protein 11
		chr3_5331914	8.5	Vitvi03g00492	0	Autophagy-related protein 2 isoform X2
		chr6_6749124	6.58	Vitvi06g00580	0	DExH-box ATP-dependent RNA helicase DExH11
		chr17_4241609	6.39	Vitvi17g00359	16 576	Partial AB-hydrolase lipase domain-containing protein
	H3	chr6_6435829	13.61	Vitvi06g00550	0	Subtilisin-like protease SBT4.4
		chr17_5317487	12.41	Vitvi17g00442	0	Transcription factor BIM2
		chr2_5711873	12.34	Vitvi02g00587	0	
		chr16_15731027	9.71	Vitvi16g00841	577	Cleavage and polyadenylation specificity factor subunit 1 isoform X2
		chr5_4003189	8.63	Vitvi05g00386	386	Uncharacterized protein LOC100259606
**pH**	H2	chr16_16737957	8.4	Vitvi16g00902	5033	
		chr2_3615910	7.82	Vitvi02g01405	2944	Thaumatin-like protein
		chr5_6565196	7.17	Vitvi05g00631	0	Sec-independent protein translocase protein TatB
		chr1_4092511	7.11	Vitvi01g00375	0	CAX-interacting protein 4
**TA**	H1	chr6_4703291	8.78	Vitvi06g00376	0	DNA-directed RNA polymerase (EC 2.7.7.6)
		chr16_16737957	6.91	Vitvi16g00902	5033	
		chr16_15872778	6.77	Vitvi16g00856	10 876	Protein kinase domain-containing protein
	H2	chr17_4852511	8.9	Vitvi17g00406	0	AP2/ERF domain-containing protein
		chr14_27817357	8.71	Vitvi14g01781	0	Protein kinase domain-containing protein
		chr6_6497421	7.32	Vitvi06g00556	10 876	UspA domain-containing protein
		chr16_16345474	7.29	Vitvi16g00889	0	Probable carboxylesterase 120
		chr16_15872778	6.77	Vitvi16g00856	5284	Protein kinase domain-containing protein
	H3	chr1_18669107	8.94	Vitvi01g01411	2469	Tricalbin-3
		chr5_3299467	6.77	Vitvi05g00328	0	CBS domain-containing protein
**Glucose**	H1	chr16_15992931	32.12	Vitvi16g00860	2963	K transporter protein
	H2	chr16_15992931	46.19	Vitvi16g00860	2963	K transporter protein
**Fructose**	H1	chr16_15992931	33.74	Vitvi16g00860	2963	K transporter protein
		chr6_6883931	6.56	Vitvi06g00591	0	Laccase (EC 1.10.3.2) (Benzenediol: oxygen oxidoreductase) (diphenol oxidase) (Urishiol oxidase)
	H2	chr16_15992931	9.5	Vitvi16g00860	2963	K transporter protein
**Malic**	H1	chr7_4063588	7.36	Vitvi07g02217	379	Avr9/Cf-9 rapidly elicited protein
	H2	chr16_15992931	30.38	Vitvi16g00860	2963	K transporter protein
		chr67346132	9.76	Vitvi06g00644	0	RING-type E3 ubiquitin transferase (EC 2.3.2.27)
		chr17_6001448	8.42	Vitvi17g00511	0	Galectin domain-containing protein
		chr1_21510649	7.22	Vitvi01g01600	4358	
**Tartaric**	H2	chr16_15992931	15.68	Vitvi16g00860	2963	K transporter protein
		chr6_11727849	14.53	Vitvi06g00907	2177	
		chr5_3947609	7.59	Vitvi05g00383	0	Procollagen-proline 4-dioxygenase (EC 1.14.11.2)
**Citric**	H1	chr17_10304589	13.51	Vitvi17g00863	0	Cysteine-rich receptor-like protein kinase 2
		chr17_13863355	6.82	Vitvi17g01087	0	Uncharacterized protein

## Discussion

### Population and phenotype adjustment

GWAS are typically performed using diverse panels of varieties. However, GWAS can also be successfully applied to multiparental and breeding populations, provided there is sufficient genetic diversity [33]. In our study, admixture analysis revealed the presence of three distinct genetic clusters, along with considerable variation both within and across populations, indicating that the population harbors substantial genetic diversity. This diversity makes it well suited for GWAS, allowing the exploitation of historical recombination events [34]. Several prior studies have effectively utilized multiparental populations in GWAS to detect marker–trait associations across various crops. For example, Jun Im *et al*. analyzed an F₂ population of 269 grape progenies to study berry weight traits [35]; Osorio-Guarín *et al*. conducted GWAS in an O × G oil palm hybrid population to examine 10 agronomic traits [25]; Liu *et al*. used a MAGIC population of 215 rice lines derived from four parents to study zinc content [36]; and Zhang *et al*. employed a population of 300 F₁ maize hybrids generated via a diallel crossing scheme to investigate yield-related traits [37]. An alternative mapping strategy often used in multiparental populations is identity-by-descent (IBD)-based mixed model QTL mapping [38]. However, in this study, we chose GWAS over IBD mapping for several reasons. Accurate haplotype phasing and IBD estimation are challenging in early-generation F₁ populations, especially when derived from interspecific crosses, due to high heterozygosity. IBD-based results can be difficult to interpret and apply in breeding programs without high-quality phased haplotype maps, which are often unavailable in complex hybrid populations [39]. In contrast, GWAS provides straightforward SNP–trait associations, enabling the identification of candidate genes and supporting marker-assisted selection in applied breeding contexts [34]. Furthermore, current GWAS employed a mixed linear model framework incorporating both population structure (Q) and kinship (K) to control for confounding effects, a well-established approach for reducing false positives in structured populations [40]. The combination of K + Q correction with stringent Bonferroni thresholding ensures the robustness of detected associations. The identification of a well-characterized QTL for berry color on chr 2 further supports the reliability of our GWAS approach [41]. The SNP chr2_13483780, located on chr 2, is in proximity to a cluster of eight Myb-related transcription factor genes (Supplementary Table S2). Previous studies have demonstrated that these Myb transcription factors regulate anthocyanin biosynthesis through activation of UDP-glucose:flavonoid 3-O-glucosyltransferase (UFGT), a key enzyme in the anthocyanin biosynthetic pathway [42]. Traditional biparental QTL mapping, which was used as an additional validation approach for the trait TSS, and the results were highly consistent with the GWAS findings. In both analyses, major QTLs were detected on chr 6, 16, and 17 (Supplementary Fig. S4; Supplementary Table S3). The effect patterns of these QTLs closely mirrored those observed in the GWAS results, with the strongest signal detected on chr 16 in H2 (GWAS –log10(p) = 52.4; QTL LOD = 7.51), followed by H1 (GWAS –log10(p) = 24.8; QTL LOD = 5.00). These findings demonstrate that GWAS performs reliably in this multifamily population and, in this case, even outperforms traditional QTL mapping in detecting major-effect loci.

In large mapping populations with multiple families, it is common for individuals to reach particular developmental stage at different times during the season. To address this limitation, we adopted a sampling design that intentionally followed a population-based phenological reference, where genotypes were sampled when the majority of individuals reached the designated BBCH stage (81, 85, or 89). This strategy was chosen to maintain synchronization with the natural ripening dynamics of the population under the same field environment, and to capture trait variation that realistically reflects genetic and developmental differences among genotypes. As a result, the number of individuals phenotyped varied across harvests and years. For example, only 155 out of 587 individuals were phenotyped during H3 in 2020, whereas 396 individuals were phenotyped during H1 in 2021 (Supplementary Table S1). To specifically control for this and other environmental variations, we did not use raw phenotype data for the final GWAS. Instead, we calculated BLUPs [43]. Crucially, as stated in the methods, BLUPs were calculated separately for each harvest by incorporating all 3 years’ data in the model. In this way, each trait has three BLUP values representing each of the three phenotyping events. This approach allowed us to generate a robust, adjusted phenotype for each developmental window (H1, H2, H3), which minimizes noise from both year-to-year environmental effects and minor developmental variations within a single harvest event. Each harvest (H₁, H₂, H₃) was analyzed independently, so associations were detected within comparable physiological windows of berry development. The reproducibility of major QTLs on chr 6, 16, and 17 across multiple harvests and years supports the robustness of this approach. The SNP on chromosome 16 showed an extremely strong association with TSS at all three time points, and its association peaked during H2, which aligns biologically with the period of rapid sugar accumulation. The same was observed when entirely different biparental QTL mapping was used as a validation for trait TSS. Trait summary statistics from Supplementary Table S1 indicate that the population exhibits substantial variability for the traits analyzed, which is desirable for association studies [44]. The population also showed moderate to high coefficients of variation (CV%) for most traits, and all three subpopulations displayed similar CV%, suggesting overlapping phenotypic diversity across subgroups. Although GWAS using raw, uncorrected phenotype data also identified most of the major associations found with BLUP-adjusted data, BLUPs tended to yield QTLs with higher significance and higher –log₁₀(p) values. Notably, some QTLs were detected using uncorrected data, including some loci previously reported in the literature were not detected in the BLUP-based GWAS. Use of BLUPs sometimes may reduce the ability to detect minor or environment-specific QTLs [45]. These minor QTLs are still biologically important, especially in traits influenced by developmental timing or environmental triggers. This masking effect of adjusted trait data has also been reported in earlier GWAS studies in grapevine [27], where the authors suggested that the use of BLUEs may have contributed to the absence of known seed weight-related QTLs that were later founded with uncorrected data. A similar phenomenon likely explains the disappearance of certain QTLs in our BLUP-based results that were present in the uncorrected data.

### Sugar-related traits

Stable QTLs for sugar-related traits were detected across all three berry developmental time points. Notably, TSS-associated QTLs on chromosomes 6, 16, and 17 were consistent across harvests (Table 1, Fig. 3). The SNP ‘chr16_15991560’ on chr16 showed strong associations with −log10(p) values of 52.4 (H2), 24.8 (H1), and 9.71 (H3). Nearby genes Vitvi16g00860 and Vitvi16g00861 encode putative sugar transporter protein that facilitates movement of sugars across cell membranes. These membrane transporters are particularly important during berry ripening, where they help in the accumulation and distribution of sugars into vacuoles of ripening berries [46, 47]. This association peaked in H2, likely reflecting heightened sugar transport as berries soften, aligning with earlier observations of rapid sugar accumulation postveraison [48]. QTLs on chr 6 fell within regions reported previously [16], with nearby genes like F6GUW5 involved in carbohydrate metabolism. QTLs detected on chr 17 coincided with genes annotated for functions such as transmembrane transporter activity, fucose metabolism, and regulation of gene expression through DNA-templated transcription. Additional QTLs were noted on chr 2 (during H2 and H3) and chr 3 (during H1 and H3), which have been reported in earlier studies [17, 20, 49]. GWAS analysis performed using the original phenotype (not BLUPs) of each year separately also identified these stable QTLs on chr 16 during every harvest event of the 3 years studied (9 out of 9 instances), QTLs on chr 6, 17, 2, and 3 were also identified in all 3 years, but missing in some harvests (6, 5, 4, and 3 instances, respectively), which may indicate their influence on TSS during specific times in the berry development (Supplementary Fig. S5). QTLs on chr 2 and 17 are mostly found during later harvests (H2 and H3), indicating their effect on the late accumulation of soluble solids in developing berries.

For glucose and fructose, a stable QTL was identified on chr 16 during both H1 and H2, with highly significant –log10(p) values (Table 1, Fig. 3). The causal SNP for this QTL is identical to the SNP previously associated with TSS and is linked to genes from the sugar transporter family, which facilitate the transmembrane movement of sugars from source to sink tissues [46, 50]. Additionally, a separate QTL for fructose was detected on chr 6 during H1. Genes in this region are involved in processes such as apoplast activity and lignin degradation in cell walls, which support substrate transport. When GWAS was performed using raw (non-BLUP) phenotypic data, several additional QTLs were identified for both glucose and fructose, including known regions on chr 14 for fructose and QTLs associated with the glucose-to-fructose ratio on chromosomes 3 and 17 [4]. These QTLs were not detected when using BLUPs, suggesting phenotypic correction might suppress these QTLs. The genetic architecture of sugar accumulation in grape berries is highly quantitative, involving >130 genes in the *V. vinifera* genome [51]. This complexity likely contributes to the detection of several strong-effect QTLs in our analysis.

### Acid-related traits

For TA, stable QTLs were identified on chromosomes 6 and 16 during both H1 and H2. The QTL on chr 6 has been consistently reported in previous studies [4, 52, 53], underscoring its robustness across different grapevine populations. Within this region, the gene Vitvi06g00376 encodes a DNA-directed RNA polymerase involved in the biosynthesis of organic acids. In addition to these stable loci, a significant QTL was detected on chr 14. The gene Vitvi14g01781, located at this locus, is associated with ATPase-binding, phosphorylation, and membrane-related functions. Prior research [54, 55] has shown that vacuolar ATPase activity, critical for proton transport across membranes, rapidly increases during ripening, facilitating oxidative phosphorylation of organic acids to meet elevated energy demands and support osmoregulation. Another nearby gene, Vitvi14g01783, located ~8 kb from this QTL, encodes a ‘SWEET’ sugar transporter, which mediates bidirectional sugar movement across membranes and may indirectly influence acid levels [56]. Additional QTLs consistent with earlier findings on chr 1 [49] and 5 [18] were also identified in this study. When GWAS was performed using raw (non-BLUP) phenotypic data, similar signals were detected on chr 6 (in five out of nine harvests), 16 (three out of nine), and 14 (three out of nine), further supporting the relevance of these loci in regulating acidity traits (Supplementary Fig. S6).

For the trait pH, a QTL on chr 1, previously reported by [20], was located within the gene Vitvi01g00375, which encodes a cation exchanger protein (CXIP4) involved in ion transport and calcium homeostasis. These functions directly influence the pH balance in grape berries. Another QTL on chr 2 is positioned near Vitvi02g01405, a gene encoding a thaumatin-like protein with ontologies related to cell defense responses. On chr 5, the identified QTL contains two genes (Vitvi05g00631 and Vitvi05g01905) that encode intercellular protein transporters, which may indirectly affect pH regulation. A QTL on chr 16, also previously identified by [20], is located near a gene with uncharacterized functional annotation. Notably, this QTL was detected in four separate instances using raw phenotypic data (Supplementary Fig. S7). All pH-associated QTLs in this study were detected exclusively during H2. This likely reflects rapid physiological changes in sugar and acid content during the berry softening stage, which strongly impact pH of the juice.

For malic acid, a highly significant QTL was identified on chr 16, with a −log10(p) value of 30.38. The nearest gene, Vitvi16g00860, encodes a membrane K^+^ transporter protein. Prior studies [53, 57] have shown that K^+^ concentration plays a key role in berry loading and regulation of acid–base balance, thereby influencing grape acidity. After veraison, grape berries become major sinks for K^+^, with concentrations increasing as ripening progresses [6]. A QTL on chr 6, also detected in this study, has been reported several times previously [4, 16, 20, 52, 53]. Within this region, the gene Vitvi06g00644 is annotated with functions related to the vacuole membrane and cytoplasm, the primary storage compartments for malic acid in berries. Another QTL on chr 17, consistent with findings from [58], have ontologies related to metal ion binding, reduction of molecular oxygen, and carbohydrate-derivative biosynthesis. These processes are involved in metal ion and sugar accumulation and may contribute to the reduction of acid content. A QTL on chr 1, also previously reported [52], was found in this study, though no ontologies were identified for nearby genes. All malic acid QTLs mentioned above were detected during H2, likely due to the rapid breakdown of malic acid during the berry softening phase. During this period, sucrose transported into the berries via the phloem is hydrolyzed into glucose and fructose [48] while malic acid is metabolized as a carbon source [59]. A QTL on chr 7 was the only one observed during H1; it is near Vitvi07g00387, a gene associated with ATP-binding and glutamine metabolism. No QTLs were detected during H3, likely because malic acid levels are largely depleted by harvest maturity, limiting related gene expression. GWAS using raw phenotypic data also identified additional QTLs on chr 3, 4, 9, and 14, in addition to the loci discussed above (Supplementary Fig. S6).

For tartaric acid, major QTLs were identified on chr 16, with the closest gene (Vitvi16g00860) also associated with several other acid and sugar traits mentioned earlier. This gene is involved in K^+^ proton and sugar antiporter activity across membranes, which contributes to the regulation of acid–base balance in berries. The stoichiometric exchange of tartaric acid protons with potassium ions leads to the formation of largely insoluble potassium bitartrate, thereby reducing free acid concentration and altering the tartrate-to-malate ratio [60]. Additional QTLs for tartaric acid were found on chr 6, consistent with previous reports [19, 52], although the nearest genes (Vitvi06g00907, Vitvi06g01800) lack characterized ontologies. A QTL on chr 5, also previously reported [49, 53], included genes related to signal transduction, carbohydrate metabolism, and chemical response. All tartaric acid QTLs were identified during H2, emphasizing the physiological importance of the berry softening stage in acid regulation. For citric acid, QTLs were detected on chr 17 (during H1 and H2), and on chr 6, 7, and 16 during H2. Additional loci were found on chr 1 and 11 during H3. Genes located near these QTLs are associated with functions such as membrane permeability, K^+^ transport, ATPase binding, and carbohydrate metabolism, mirroring trends seen in other acid-related traits. To our knowledge, no previous QTLs have been reported for citric acid in grape, suggesting these findings may be novel.

### Berry mass

Genetic control of berry mass was examined by identifying QTL regions associated with single berry mass, which revealed a stable and previously reported QTL region on chr 11 consistently detected across all three harvests [17, 20, 26]. The nearest gene to this SNP Vitvi11g00092 encodes a growth-regulating protein (D7TCG2) involved in organ development. A significant QTL on chr 18 was also observed during H1, aligning with earlier findings [28]. This locus, located within Vitvi18g01924, is linked to cytoskeleton organization and protein catabolism, processes likely influencing berry weight. Notably, this hit on chr 18 ~ 1.2 Mb from Vit_18s0041g01880 (VviAGL11), a MADS-box transcription factor gene widely known for its role in seed development [41, 61]. Additional QTLs were identified on chr 4 and chr 6. The chr 4 QTL is proximal to Vitvi04g01831, which encodes the auxin-responsive protein SAUR72, a regulator of fruit ripening and growth [5]. On chr 6, the QTL overlaps with Vitvi06g00051, a gene producing tubulin alpha chain proteins essential for mitotic cell cycle and microtubule cytoskeleton organization. A QTL on chr 17, previously noted in multiple studies [26, 62], was also detected in this study during H3, reinforcing its potential role in berry mass regulation.

### Gene ontologies

GO analysis of loci surrounding significant SNPs provided valuable insights into the functional relationships between traits, aiding in the interpretation of their genetic architecture. Shared GO terms among different traits suggest overlapping genetic mechanisms, thereby helping to link candidate genes to multiple phenotypic traits. This strategy has previously been employed in grape breeding and in other domains such as human disease genetics [27].

In this study, stronger correlations were observed within the CC–GO subset across all traits (Fig. 6), likely due to the generalized role of cellular structures, particularly membranes, which are fundamental to the function of all quantitative traits analyzed. Membrane-associated GO terms were the most frequently observed in this subset, consistent with the critical role of membrane transport in sugar and acid accumulation. In contrast, the MF–GO and BP–GO subsets, being more trait-specific, exhibited a smaller number of shared ontologies between traits that resulted in lower overall correlations (Fig. 6), a similar observation was seen a previous study [27]. However, these subsets offered deeper functional insights due to their specific nature. Within the MF–GO subset, the most frequently shared functions among sugar and acid traits included potassium (K^+^) antiporter activity, ATP binding, and ATP hydrolysis. As discussed earlier, these processes are essential for ion transport and energy regulation, particularly after veraison when K^+^ redistribution from leaves to berries increases dramatically [63]. K^+^ plays a key role in maintaining the transmembrane potential, which governs the uptake of sugars and other solutes [5]. In the BP–GO subset, phosphorylation and protein glycosylation were among the most prevalent biological processes, especially shared among TSS, malic acid, tartaric acid, citric acid, and berry mass traits. Protein phosphorylation, mediated by kinases, is critical for cell signaling pathways and is likely a key regulator of berry development [64].

## Conclusion

In this study, highly significant marker–trait associations were identified for nine fruit quality traits observed. A total of 52 distinct SNPs were found to influence trait phenotypes. Among these, 31 SNPs were located within genes, while the remaining 19 were situated within 5 kb of the nearest gene. These significant regions include both novel associations and previously reported QTLs from the literature. A particularly strong and novel QTL was detected on chr 16, associated with all sugar- and acid-related traits. The nearest genes to this region, Vitvi16g00860 and Vitvi16g00861, are involved in the transport of sugars and K^+^ ions across membranes. This function facilitates sugar accumulation and the neutralization of organic acids through K^+^ ion influx during berry ripening. Another major QTL was identified on chr 6, associated with all acid traits, TSS, fructose, and SBM. Genes in this region are involved in transmembrane transporter activity, carbohydrate metabolism, and signaling pathways responsive to environmental stimuli. This QTL has also been reported in previous studies for its role in berry acid regulation, and the current findings further validate its significance across different populations. On chr 17, QTLs associated with TSS and all acid-related traits, except tartaric acid, were identified. Genes in this region are linked to phosphorylation, ATPase activity, and membrane-related functions, all of which can indirectly influence acid metabolism in berries. These three major QTL regions on chromosomes 6, 16, and 17 were consistently identified across multiple harvests, underscoring their stability over different stages of berry development. For SBM specifically, a stable QTL was consistently detected on chr 11 across all three harvest time points. Additionally, QTLs were identified on chromosomes 1–5, 7, 14, and 18 for various fruit quality traits across different harvests. The study also emphasized the value of phenotyping at multiple ripening stages to better capture variation during berry development. Moreover, it highlighted the importance of data handling methods, as the use of BLUP-adjusted values appeared to mask several minor QTLs. Functional annotation using GO helped link candidate genes to their roles in the observed traits. Overall, these GWAS findings contribute to a deeper understanding of the genetic architecture underlying major sugar and acid traits in interspecific grape populations. The results provide a valuable foundation for molecular breeding aimed at improving fruit quality traits.

## Materials and methods

### Plant material

A total of 1064 segregating F1 individuals were created by crossing three different selections from the NDSU GGEP in a half-diallel fashion. Many of these individuals failed to produce fruit within the study time frame or succumbed to severe abiotic and biotic stresses. Ultimately, 587 well-established genotypes were used for this study. This population consists of three distinct families formed through the crossing of three internal breeding lines, ND.213 (C16 × ‘Alpenglow’), SKND.009.41 (*V. riparia* L × ‘Csaba Gyöngye’), and ND.054.27 (‘Frontenac gris’ × ‘Adalmiina’) in a half-diallel without reciprocal crosses (Supplementary Table S4) The crossing design and genetic background of the three parental lines were presented in Supplementary Fig. S8. These crosses were performed during the year 2016, and resulting seedlings were raised in greenhouse conditions in the winter and spring of 2017 before transplanting them in the summer of 2017 in a research vineyard located at NDSU Agriculture Experiment Station, Fargo, ND, USA (46°53′28.9″N 96°48′46.9″W). All vines were trained on to a single wire (1.52 m height) unilateral, spur-pruned cordon with row-to-vine spacing of 0.91 m within row (N–S) and 1.5 m between rows (E–W). Drip irrigation and standard agronomic practices were followed to maintain the vines.

### Phenotyping

Phenotypic characterization of fruiting individuals within the population was performed for 10 fruit traits: TSS, pH, total acidity (TA), glucose, fructose, malic acid, tartaric acid, citric acid, yeast-assimilable nitrogen (YAN), and SBM for three consecutive years (2020–22). During each of the 3 years, phenotypic evaluations were conducted at three distinct time points as three different phenotyping events for a total of nine fruit phenotyping events. These time points were decided based on visual Biologische Bundesanstalt, Bundessortenamt, and Chemical industry (BBCH) phenological stage ratings for the majority of the individuals undergoing phenotyping. For ease of communication, phenotyping events from harvest one, two, and three are denoted as H1, H2, and H3, respectively. During H1, phenotypic data were collected from vines based on the majority of the population achieving BBCH growth stage 81, which marks the onset of ripening when berries begin to develop color. During H2, the majority of individuals approached BBCH stage 85, indicating berry softening. In H3, phenotyping was conducted when most of the individuals achieved stage 89, which corresponds to full ripeness, when berries are ready for harvest. The number of individuals phenotyped during each event varied across harvests and years, primarily due to fluctuations in the number of fruiting vines each season and the requirement that individuals be at a specific developmental stage during phenotyping (Supplementary Table S1). As a result of these limitations, only 482 out of the 587 genotypes were phenotyped across the study period. During H1 and H2, 20–30 berries were randomly sampled from each grapevine. During the final evaluation time point within a year (H3), a total of 60–70 berries were sampled depending on fruit availability. Fruit was collected and transported from the field to the lab where it was immediately processed for evaluation.

Berry mass was measured by dividing the mass of each sample by total number of berries within the sample. Juice was extracted from the collected samples by manually crushing the berries within plastic sampling bags. Traits were measured from the extracted juice such as TSS using a Pal-1 digital refractometer (Atago Co., Tokyo, Japan), pH using ATAGO PAL-pH handheld digital pH Meter (Atago Co., Tokyo, Japan), and total acidity was measured with a PAL-BX|ACID5 grape brix-acidity meter (Atago Co., Tokyo, Japan). The results were measured as a percentage (grams of malic acid equivalent/100 g). Further characterization of musts was performed via high-performance liquid chromatography (HPLC) on a reserved juice sample of ~5 ml to quantify glucose (g/l), fructose (g/l), malic acid (g/l), tartaric acid(g/l), and citric acid (g/l) content. For the first year (2020) samples, HPLC analysis was performed at the Midwest Grape and Wine industry Institute at Iowa State University (Ames, IA, USA) using an Agilent 1200 series HPLC (Agilent Technologies, Inc., Santa Clara, CA, USA). For later years (2021 and 2022), HPLC analysis was performed at Northern Crop Institute (NCI, Fargo, ND, USA) using Agilent 1260 Series Infinity II HPLC (Agilent Technologies, Inc., Santa Clara, CA, USA). Must YAN was quantified using enzymatic kits to assess total primary amino nitrogen (K-PANOPA, Neogen, Lansing, MI, USA) and ammonia (K-AMIAR, Neogen, Lansing, MI, USA) with readings recorded using a Biotek Cytation5 plate reader (Agilent Technologies, Inc., Santa Clara, CA, USA) and a UV–vis spectrophotometer (Genesys 10S UV–VIS spectrophotometer, Thermo Scientific, NY, USA), respectively. During the 2020 season, HPLC traits were measured only for samples collected at final harvest (Supplementary Table S5). In all 3 years, YAN was measured only in final harvest samples. Must YAN was later excluded from GWAS analysis due to poor consistency of data.

Anthocyanin content using berries from the population was also measured over 2 years (2022 and 2023) to evaluate berry color loci, a well-established trait in grapes that aids in assessing the model performance. For this purpose, a total of 50 frozen berries per genotype were thawed at room temperature prior to anthocyanin extraction. Once fully thawed, the berries were transferred to a plastic container and homogenized into a uniform paste using a high-speed homogenizer (Ultra-Turrax T25, IKA Works, Inc., Wilmington, NC, USA) operating at 24 000 rpm for 45–60 s. The homogenate was mixed thoroughly, and 1 g of the paste was weighed into a 15-ml centrifuge tube. Subsequently, 10 ml of 50% (v/v) aqueous ethanol (pH 2.0) was added, and the tube was gently inverted every 5–10 min over the course of 1 h to ensure efficient anthocyanin extraction. Following extraction, samples were centrifuged at 3500 rpm for 5 min (LC-85000, Benchmark Scientific, New Jersey, USA). The resulting supernatant was carefully collected as the anthocyanin extract. Total anthocyanin content in the berry extracts was quantified using the hydrochloric acid method [65], which measures the characteristic absorbance peak of anthocyanins at 520 nm. Because anthocyanins exhibit enhanced red coloration under strongly acidic conditions, the extracts were diluted with 1 M HCl to promote conversion to the red flavylium ion form [66]. Specifically, 1 ml of extract was transferred into a 15-ml tube, diluted with 10 ml of 1 M HCl, and allowed to stand for 3 h. Each sample was analyzed in triplicate. For each replicate, 374 μl of the diluted extract was dispensed into a 96-well plate, and absorbance at 520 nm was recorded using a microplate reader (Spectro-nano, BMG Labtech, Cary, NC, USA). Total anthocyanin concentration was expressed as milligrams of anthocyanins per gram of berry weight.

Before performing GWAS analysis a linear mixed model was fit using the lmer function from lme4 package by taking year and genotype as random effects to calculate BLUPs to eliminate seasonal bias. The general statistical model can be expressed as


\begin{equation*} Y_{ijk}=\mu+u_i+v_j+e_{ijk} \end{equation*}

where *Y_ijk_* represents measurement for trait of the *k*^th^ observation. *μ* is overall population (intercept) mean for the trait. *u_i_* is the random effect associated with *i*th year on the trait measurement. *v_j_* is the random effect of the *j*th genotype. *e_ijk_* is the random error associated with the *i*th year and the *j*th genotype for the *k*th observation. Since three different harvests in each year were performed at different developmental stages of the berries. We calculated BLUPs separately for each harvest by incorporating all 3 years’ data in the model. In this way each trait has three BLUP values representing each of the three phenotyping events. This allowed us to perform GWAS for each harvest event separately to assess the genetic basis for the traits at different time points in fruit development.

### Genotyping and quality control

For Genotyping-by-Sequencing (GBS) marker assessment, fresh leaf tissue samples were collected from all genotypes for DNA extraction. Collected samples were immediately lyophilized by freeze drying to −80°C using Labconco freeze drier (Labconco, Kansas City, MO, USA). Lyophilized plant tissues in 2.2-ml deep-well plates were ground into a fine powder using a Retsch mixer mill (Retsch, Haan, Germany). Following homogenization, 500 μl of extraction buffer was added to each well, and the plates were incubated at 65°C for 30 min with brief vortexing every 10 min. After incubation, the plates were placed in a −20°C freezer for 10 min. Subsequently, 166 μl of protein precipitation solution was added to each well, and the plates were incubated again at −20°C for 10 min. The plates were then centrifuged at 4000 rpm for 25 min to pellet the debris. From the resulting supernatant, 400 μl was transferred into a fresh 2.2-ml AcroPrep™ ultra purification filter plates (Cytiva, USA) containing 600 μl of DNA-binding solution per well. These filter plates were centrifuged at 3000 rpm for 10 min to allow the solution to pass through the membrane. To wash the bound DNA, 800 μl of wash solution was added to each well and the plates were centrifuged at 3000 rpm for 5 min. This wash step was repeated twice. Finally, ~200 μl of elution buffer was added to each well and centrifuged at 3000 rpm for 10 min. The elution step was also performed twice to ensure maximum DNA recovery. Restriction enzymes PstI-MseI were used to prepare DNA libraries as per the GBS protocol [67]. Sequencing of prepared libraries was conducted using Illumina HiSeq 2000 at the University of Texas Southwestern Medical Center (Dallas, TX, USA). Short reads generated from Illumina sequencing were aligned to the grape reference genome PN40024 12X.v2 assembly using Bowtie2 aligner. SNP calling was completed using Tassel GBSv2 pipeline [68]. Tassel 5.2 software was used to filter out genotypes with >10% missing data and minor allelic frequency (MAF) <0.05. After filtering SNP data, a total of 19 518 GBS markers remained. Finally missing values were imputed via the LD-kNNi method in Tassel 5.2.

Traditional biparental QTL mapping was conducted for one of the traits evaluated in this study to benchmark the GWAS results and assess the applicability of association analysis using multifamily populations such as half-diallels, rather than relying solely on diverse germplasm panels. For this analysis, we utilized the genetic map previously developed for Population 2 using rhAmpSeq markers. Genomic DNA used for rhAmpSeq marker development was extracted by Intertek AgriTech (Alnarp, Sweden) and processed without normalization to target core genome haplotypes following the protocol described by Zou *et al*. [42]. Marker amplification (half-reactions of the rhAmpSeq Library Kit; IDTDNA, Redwood City, CA, USA), indexing, pooling, purification, and Illumina sequencing (2 × 150 bp) were performed at Cornell AgriTech (Cornell University, NY, USA) for local haplotype analysis and quality filtering as described in prior studies [42, 69]. Before genetic map construction, quality control was performed by importing the VCF file for Population 2 into TASSEL. Genotypes were imputed using the LD-kNNi method with default parameters, and markers with >95% missing data were removed. Multidimensional scaling (MDS) analysis was then applied to identify and correct Mendelian errors, which helps eliminate accidental self-pollinations and unintended crosses. Genetic mapping was carried out using Lep-MAP3 v0.2 (LM3), following sequential module implementation: parental genotype calling, removal of distorted markers, assigning markers to the 19 grape chromosomes, and ordering markers within linkage groups [70]. The resulting map was then imported into the ‘R/qtl’ package [46] and formatted as an F2 cross (genotype codes assigned as AA = 1 1; AB = 1 2 or 2 1; BB = 2 2). Finally, the syntenic relationship between the genetic and physical maps was evaluated by plotting genetic positions against physical coordinates based on the *V. vinifera* PN40024 12X.v2 reference genome [71].

### Population structure and LD analysis

Population structure was determined by ADMIXTURE program using the filtered marker set by running 1000 iterations for K values ranging from 1 to 10 to generate admixture proportions. The best value of K from the generated admixture was determined by cross-validation error and log-likelihood estimations. Cross-validation error output was used to plot the k-value pattern using R studios. LD between marker sites was assessed using Tassel 5.2 software. LD was measured using standardized disequilibrium coefficients (D′) and squared allele-frequency correlations (*r*^2^) for locus pairs. Only sites where the frequency of the rarer allele was at least 0.05 were included, as D′ and *r*^2^ exhibit large variances with rare alleles [72]. The decay of LD with the physical distance (in base pairs) between sites within the same candidate locus was analyzed using a nonlinear regression model with an arbitrary recombination fraction (C) value of 0.1 within R studio.

### Genome-wide association study

Association analysis between nine different fruit traits and the 19 518 marker sets were performed for all three harvests to identify significant marker–trait associations. Blink model in GAPIT3 package [73] was utilized to conduct the GWAS analysis through implementation in R statistical software. The first three Principal Components (PCs) along with kinship were incorporated into the model to prevent spurious associations by accounting population structure into the model as supported by ADMIXTURE analysis. To reduce the false positives (Type 1 error), a stringent significant threshold cut-off using Bonferroni correction at 0.01 level (–log_10_P = 6.32) was adopted. Quantile-Quantile (Q-Q) plots were created to visualize deviations of observed *P*-values from the null hypothesis to monitor for underlying confounding effect. To evaluate the performance of the selected GWAS model, we tested it using anthocyanin content as a representative trait, applying the same parameters used for the other nine traits. Anthocyanin content was chosen because several previous studies have consistently identified stable loci associated with berry color.

Traditional QTL mapping was carried out for trait TSS in the ‘R/qtl’ environment using the F2-cross formatted genetic map with 697 rhAmpSeq markers and default interval mapping parameters. Genotype probabilities were estimated along the linkage map using the Kosambi mapping function with a 5 cM step size. Composite interval mapping (CIM) was implemented via the *cim* function to identify QTLs. To establish significance thresholds for LOD scores, 1500 permutation tests were performed, applying an alpha level of 0.10 to enable the detection of minor-effect QTLs.

### Gene annotation and gene ontologies

Gene annotation of significant regions was done with the help of annotated PN40024 12X.v2 reference genome ‘.gff’ file from Grapedia. Regions with exons and introns were excluded to keep only genes from the reference file. Bedtools were used to extract the genes that intersect significant SNPs from GWAS. To achieve this, GWAS hits were sorted into bed format with the first column denoted as chromosome and the second and third column as the start and end position of the SNP, respectively. Additional columns containing relevant information to the study were also included. The start and end positions of the SNPs were extending 20 kb in both directions to include 40-kb region around the SNP. After creation of bed files, the bedtools intersect function was used to search genes in the defined region around each GWAS hit. Finally, Blast2GO annotations from Grapedia were used as functional annotation of extracted genes for better understanding.

The physical location of significantly associated markers of each trait were used as an indicator to identify potential candidate genes. Based on LD patterns of the population, a bin of 20 kb around the marker location, which corresponds to ***±***10 kb from the SNP, was selected as a conservative threshold to avoid false positives. If no genes were identified within the defined region, that SNP was considered as missing a candidate gene. For all identified candidate genes, GO information was extracted from Uniport database. Usually there are multiple GO terms for each gene. Based on hierarchy, these GO terms were classified into three subcategories: cellular function, molecular component, and biological process. GO terms obtained for the candidate genes under significant SNPs were used to establish a functional relation between traits by performing network correlations based on shared GOs. A GO term frequency table (GO term × trait) was generated, and this matrix was used to calculate pairwise Pearson correlations between traits based on shared GOs. Strength of the correlation was determined depending on number of shared GO terms. Particularly, trait specificity in terms of contribution toward various GOs was observed by comparing frequent GO terms that appeared more than twice (Fig. 6). The data analyses were performed in R version 4.3.3 using packages dplyr for data processing, and ggplot2, CMplot, and igraph for data visualization.

## Supplementary Material

Web_Material_uhaf353

## Data Availability

The data underlying the findings presented in this article are available in the Figshare repository. The phenotype (https://doi.org/10.6084/m9.figshare.29299808.v1), genotype (https://doi.org/10.6084/m9.figshare.29300051.v1), and the QTL genetic map data files (http://doi.org/10.6084/m9.figshare.30494765.v1) can be accessed using assigned DOIs.
