# Supplementary material for: Dissecting the genetic basis of relevant fruit quality traits in interspecific grapevines (Vitis spp.)
Source: Hortic Res. 2025 Jan 6;13(4):uhaf353. doi: 10.1093/hr/uhaf353 (PMC13091398; doi:10.1093/hr/uhaf353)
Supplement: Web_Material_uhaf353 [file web_material_uhaf353.zip › Supplementary Figures.docx]

Supplementary Figures


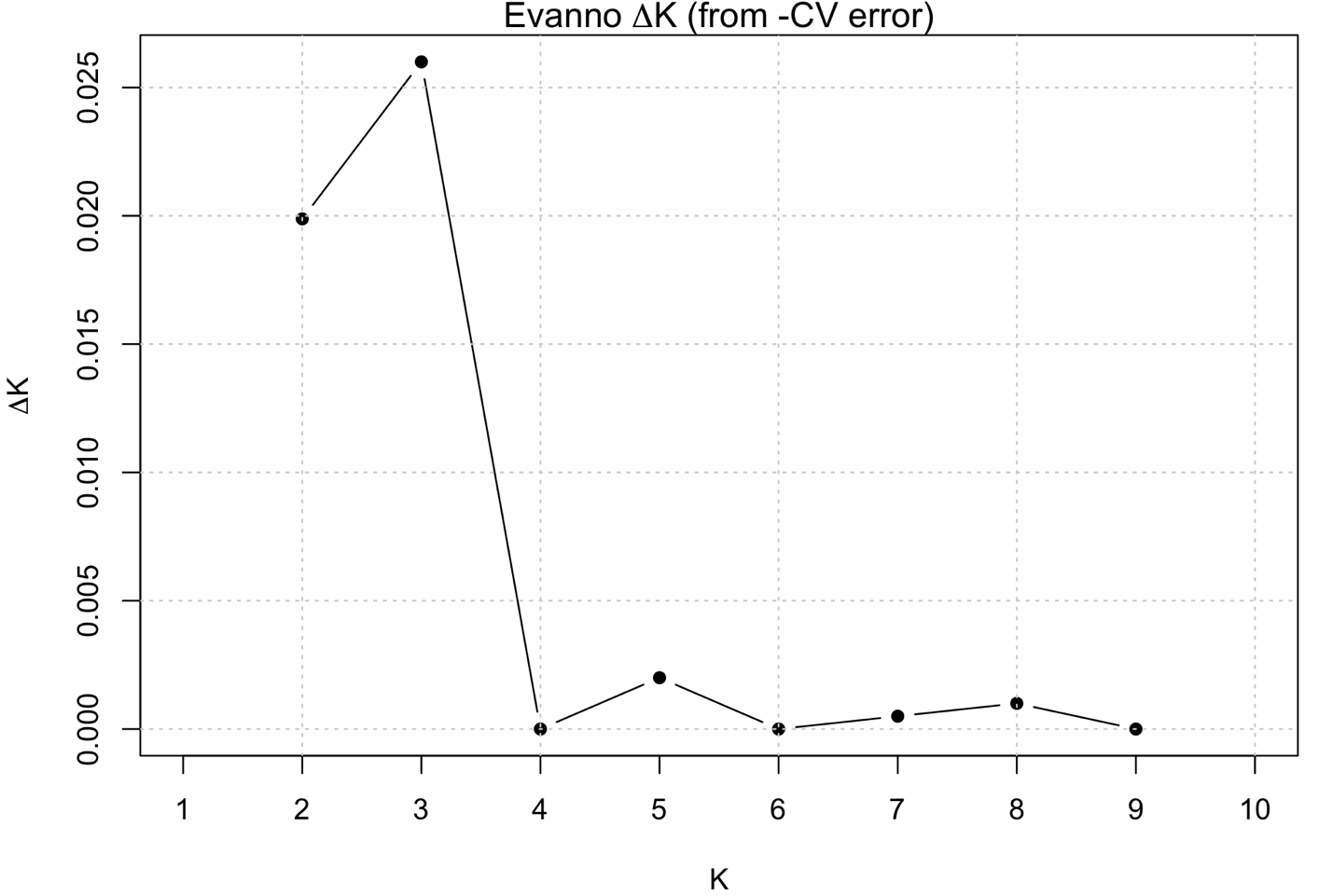


**Supplementary Figure. S1** Evanno ΔK values showing the rate of change in log-likelihood between successive K values


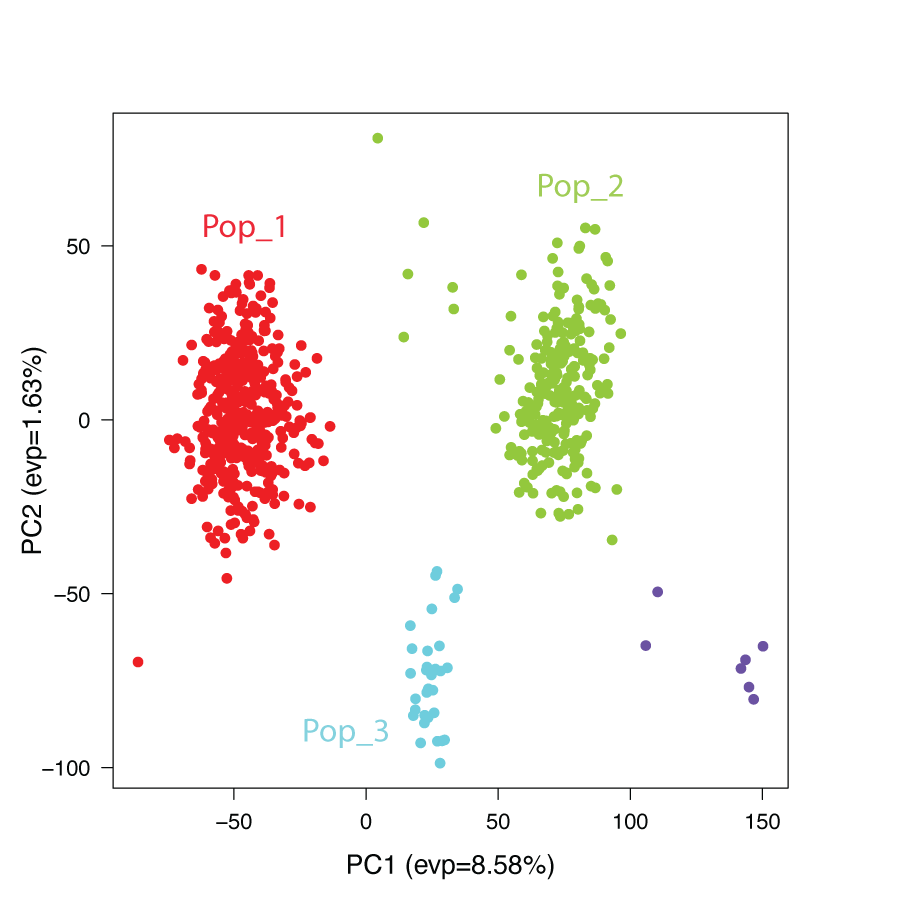


**Supplementary Figure. S2** Principal component analysis plot based on marker data showing population structure


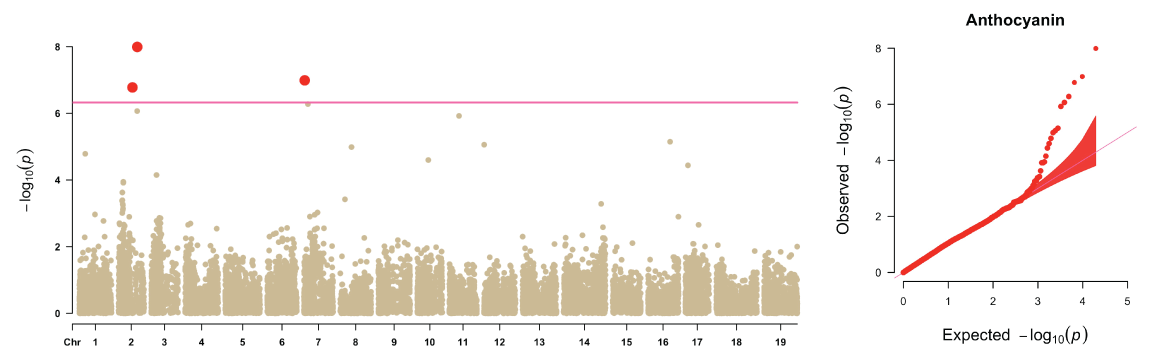


**Supplementary Figure. S3** Manhattan plot and Q-Q plots of anthocyanin content in the population. The pink vertical line denotes significant threshold at Bonferroni correction of 0.01 [-log_10_(p) = 6.32].


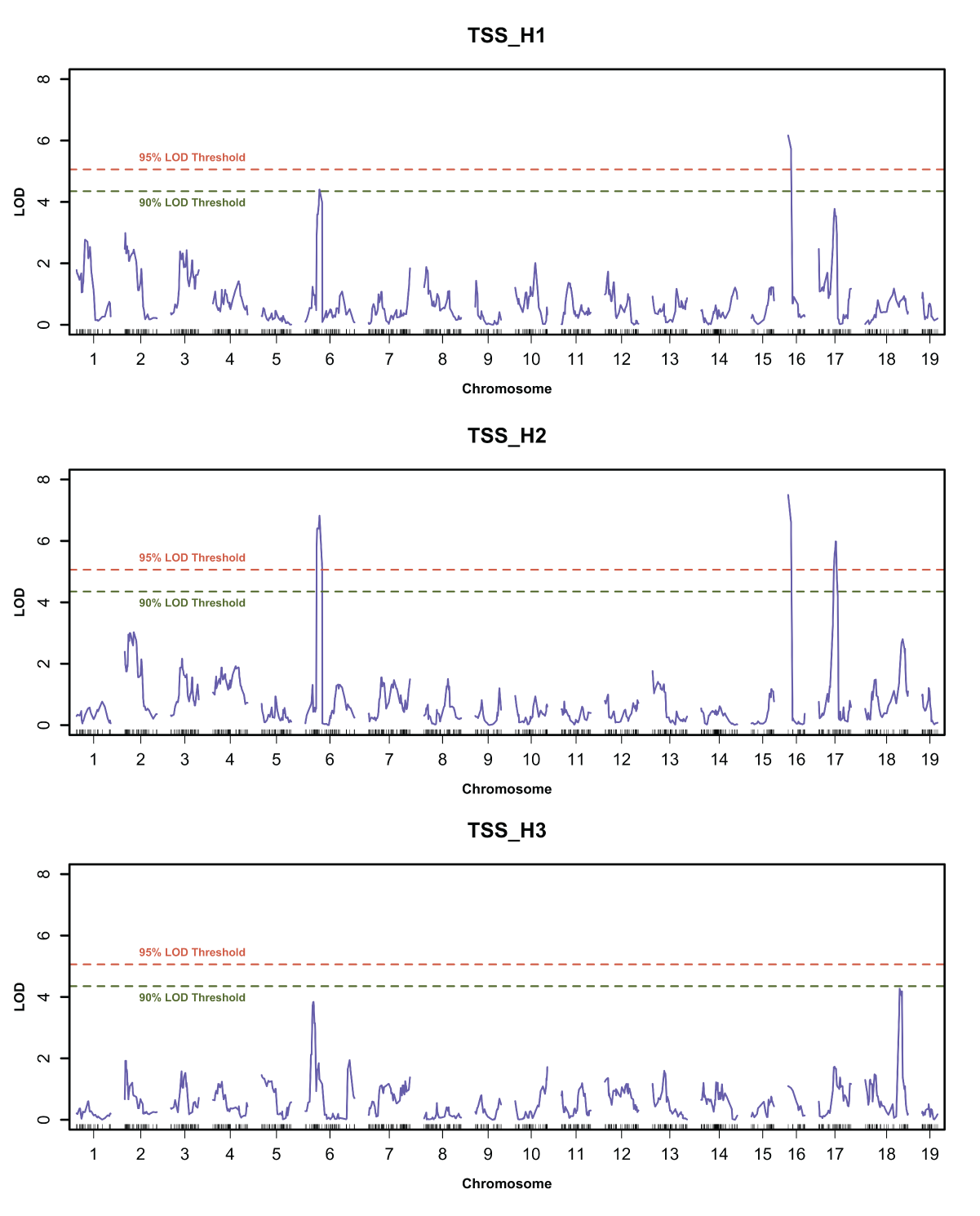


**Supplementary Figure. S4** Results from the Bi-parental QTL mapping in population 2 for the trait TSS showing the significant QTLs across three harvests.


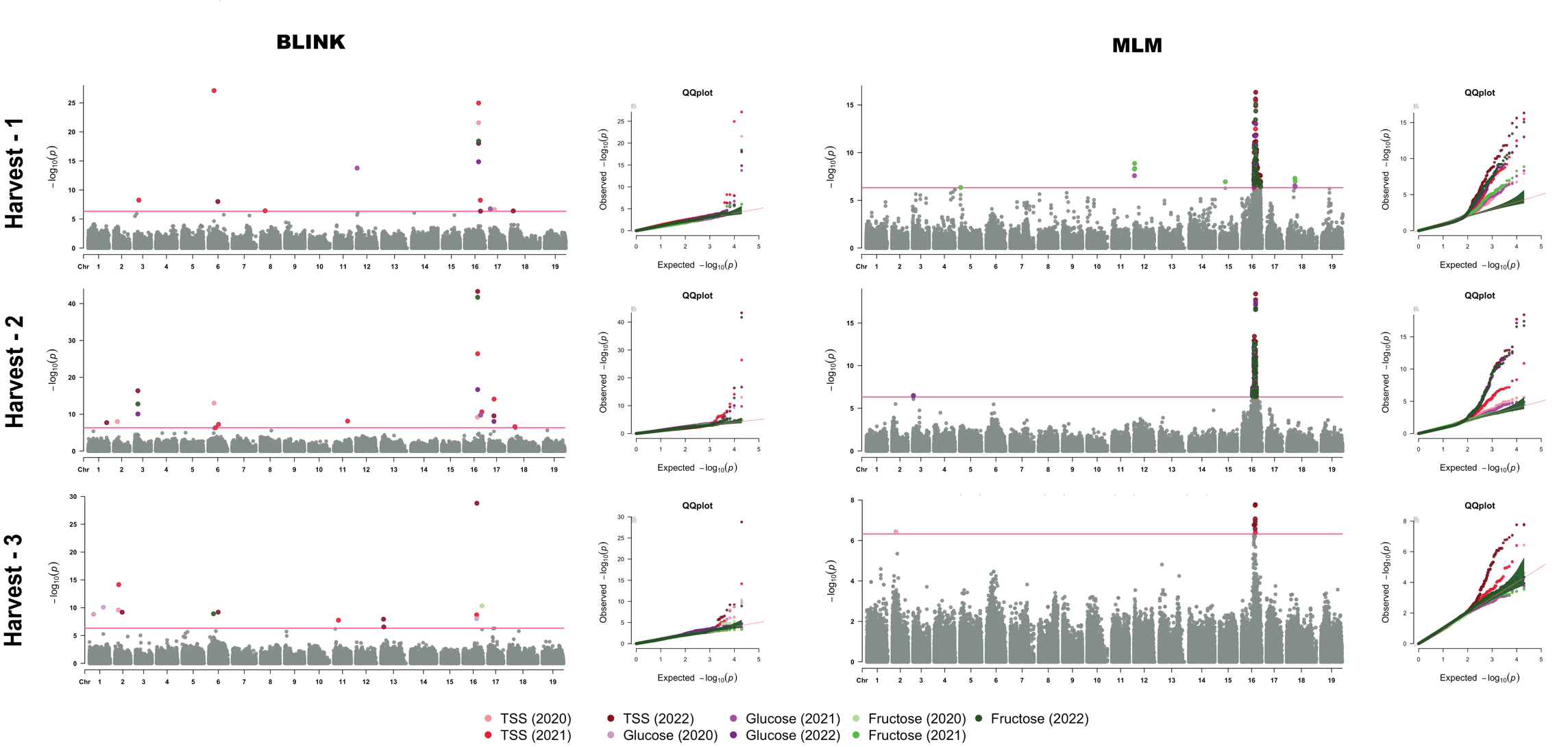


**Supplementary Figure. S5** Manhattan and Q-Q plots of sugar related traits using original phenotype. Left side panel is showing the results using BLINK model whereas right side panel showing results using MLM model. From top to bottom plots belongs to harvest one to three respectively.


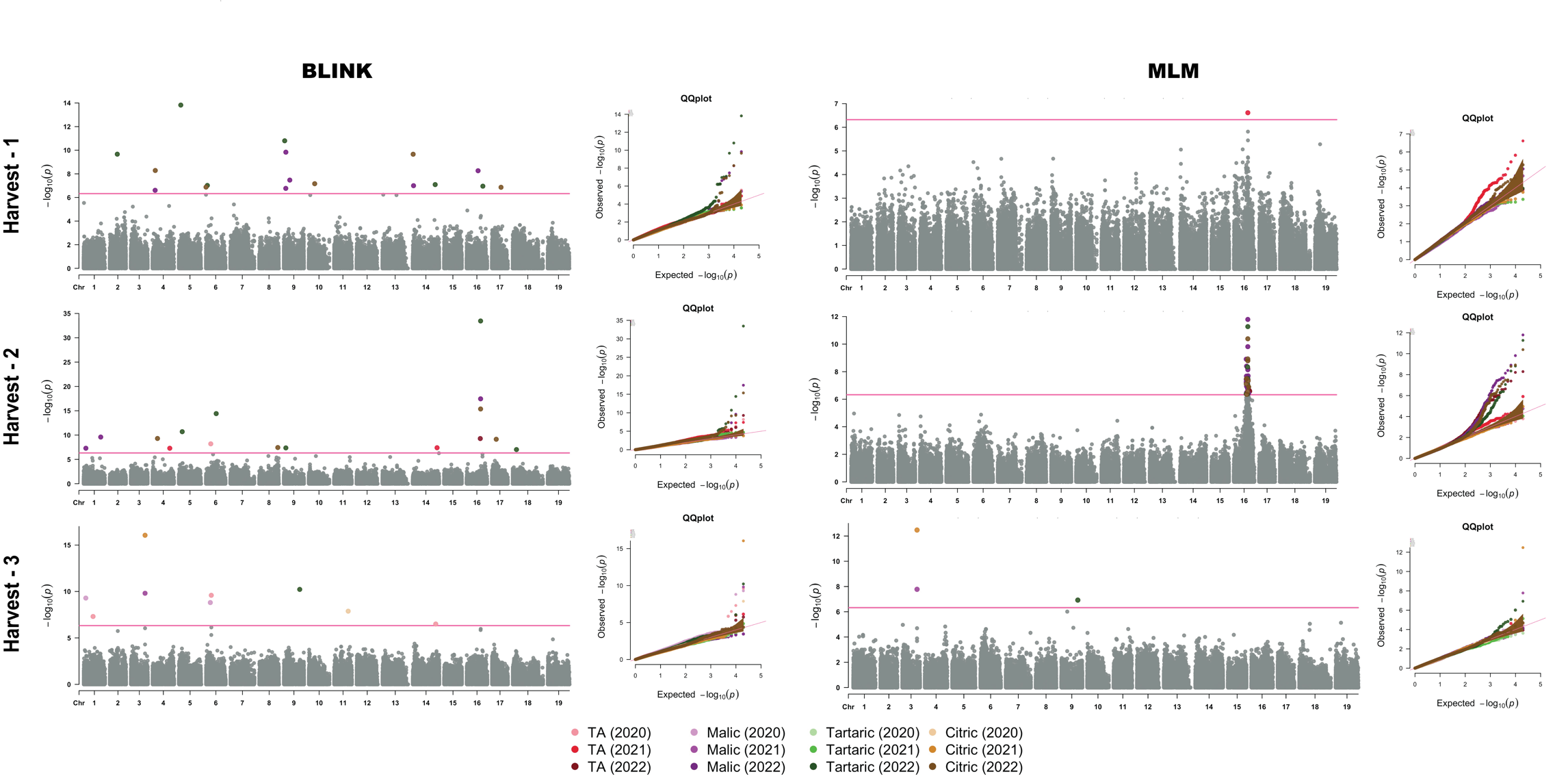


**Supplementary Figure. S6** Manhattan and Q-Q plots of acid related traits using original phenotype. Left side panel is showing the results using BLINK model whereas right side panel showing results using MLM model. From top to bottom plots belongs to harvest one to three respectively.


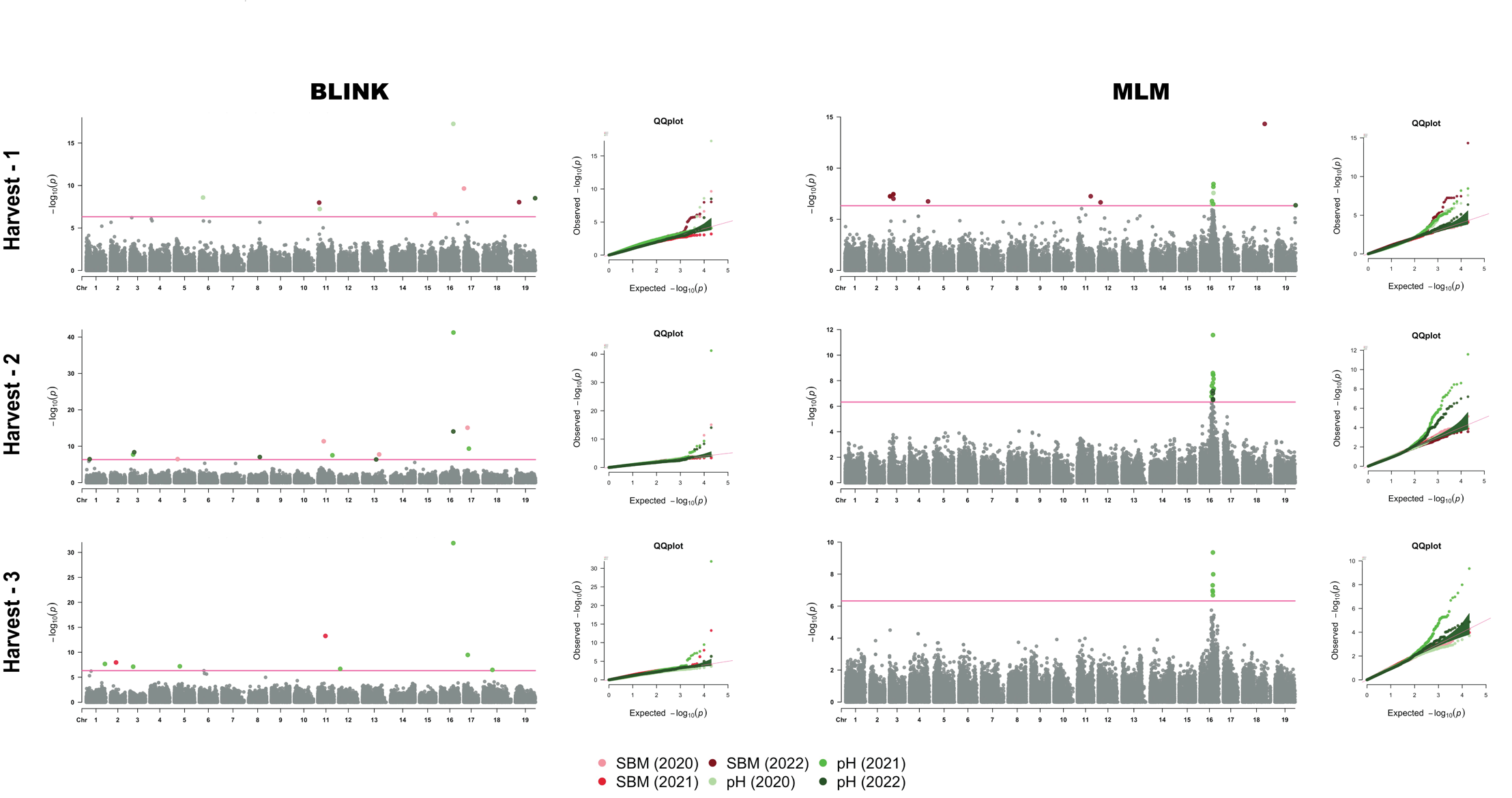


**Supplementary Figure. S7** Manhattan and Q-Q plots of traits SBM and pH using original phenotype. Left side panel is showing the results using BLINK model whereas right side panel showing results using MLM model. From top to bottom plots belongs to harvest one to three respectively.

**
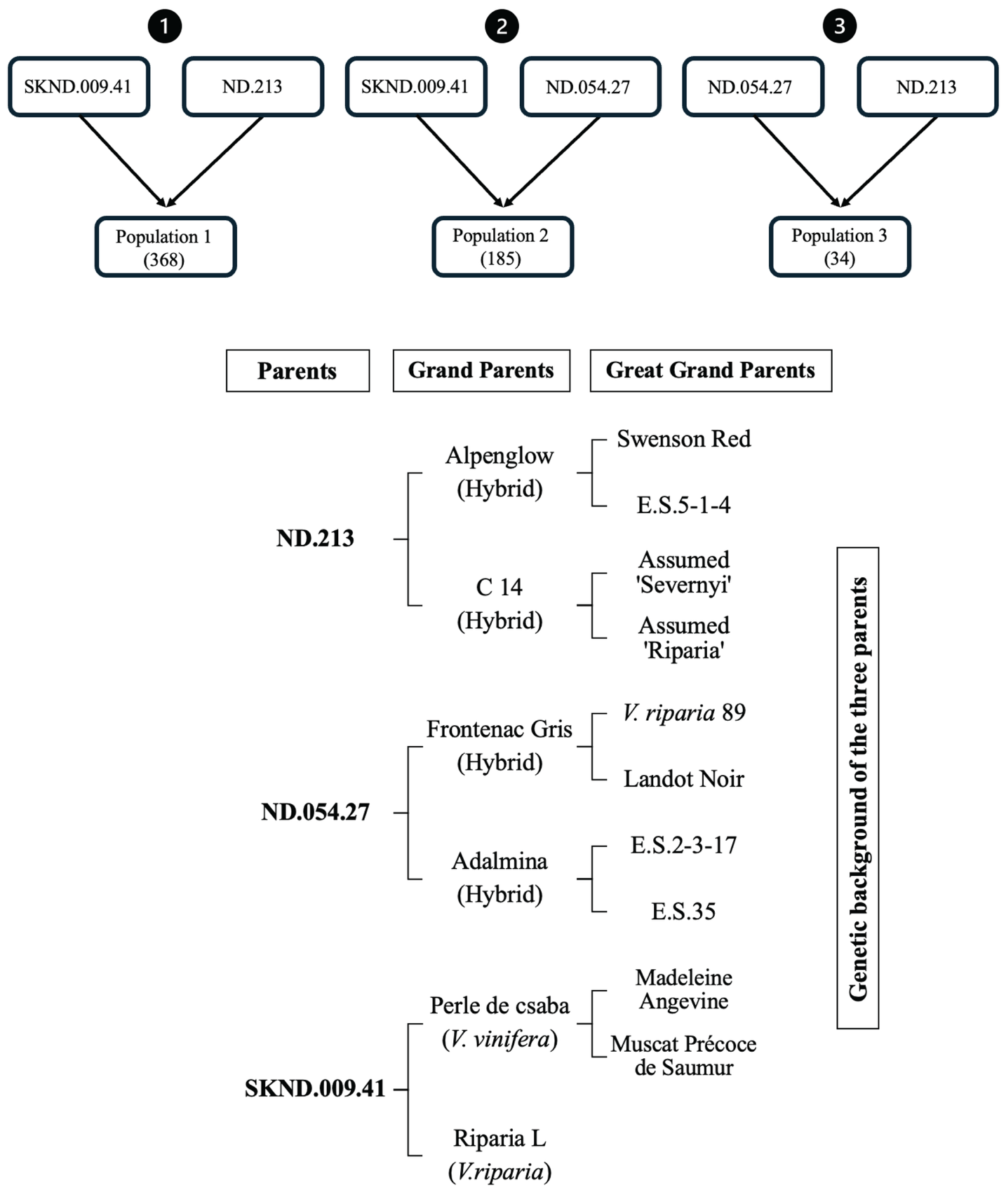
**

**Supplementary Figure. S8** crossing design used to create three distinct populations from three different parents and their genetic background.
