# Supplementary figures and images for: Dissecting the genetic basis of relevant fruit quality traits in interspecific grapevines (Vitis spp.)
Source: Hortic Res. 2025 Jan 6;13(4):uhaf353. doi: 10.1093/hr/uhaf353 (PMC13091398; doi:10.1093/hr/uhaf353)

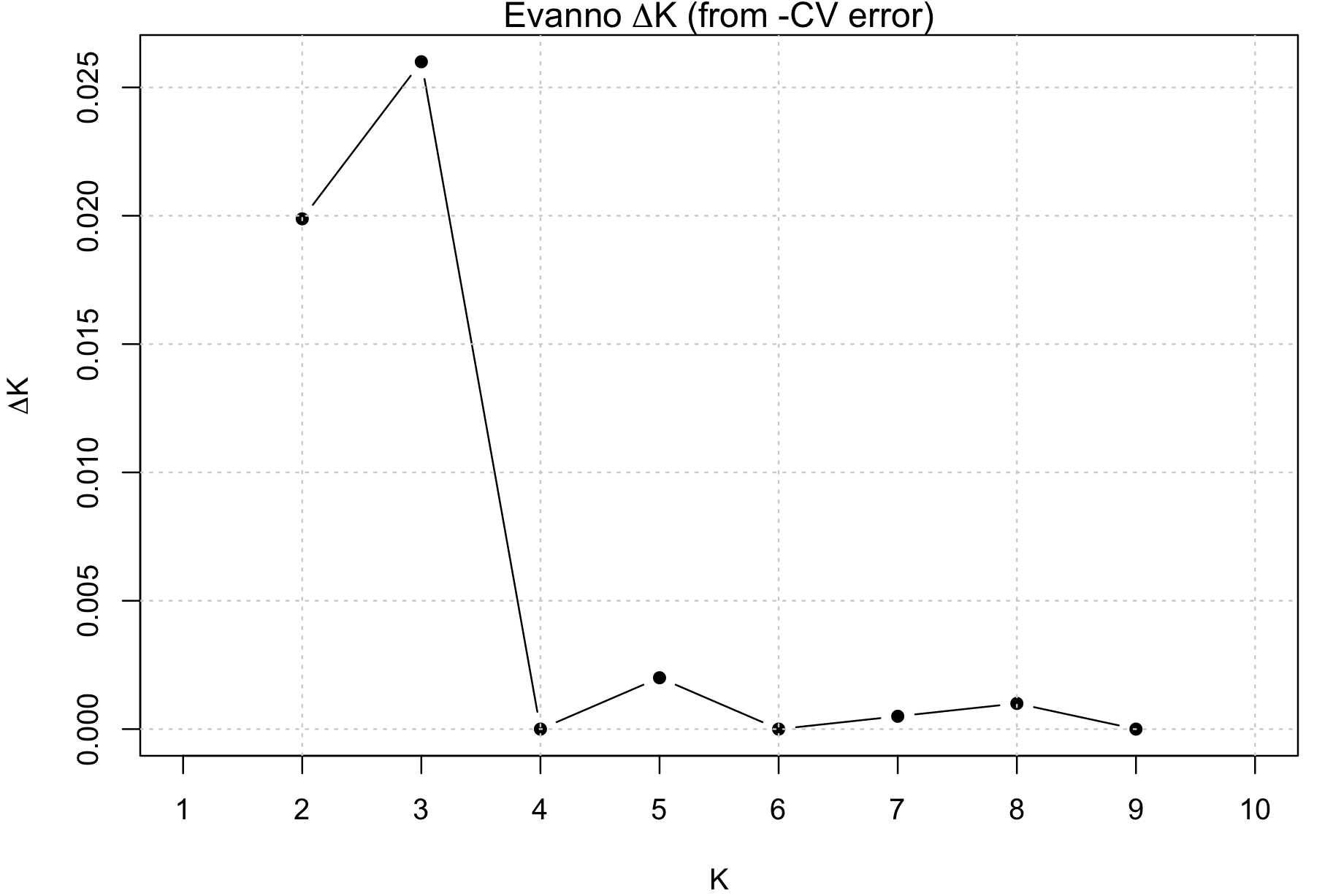

Supplement: Web_Material_uhaf353 [file web_material_uhaf353.zip › Sup_fig_S1.png]

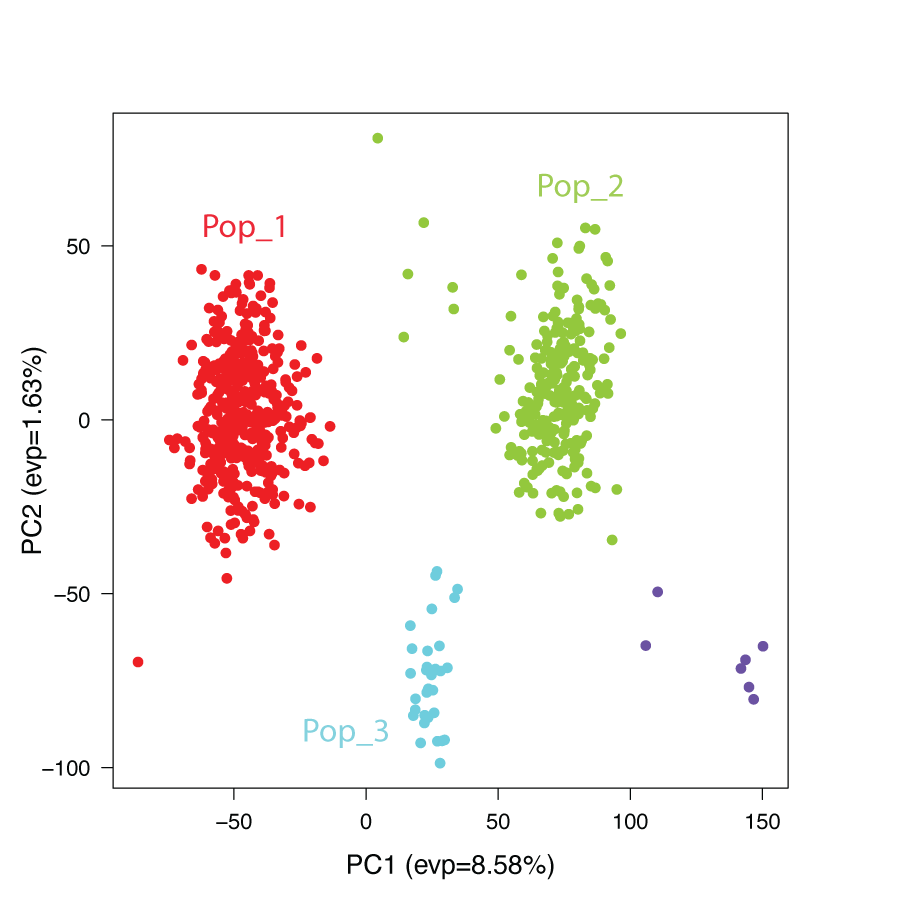

Supplement: Web_Material_uhaf353 [file web_material_uhaf353.zip › Sup_fig_S2.png]

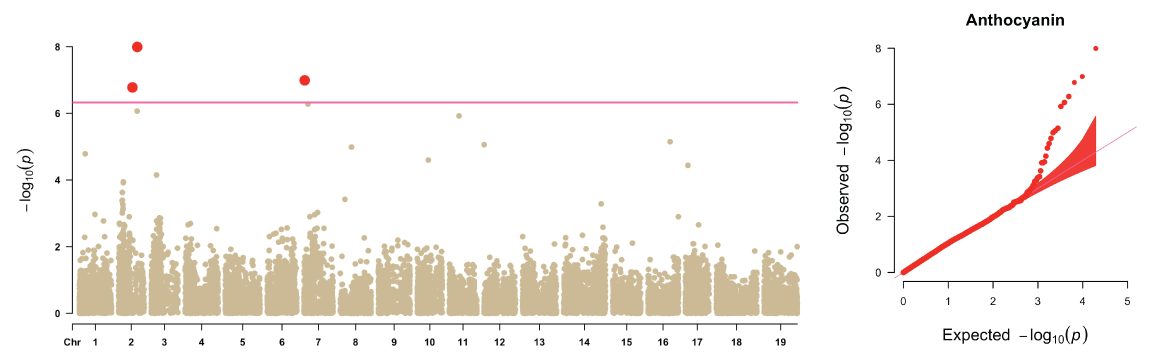

Supplement: Web_Material_uhaf353 [file web_material_uhaf353.zip › sup_fig_S3.png]

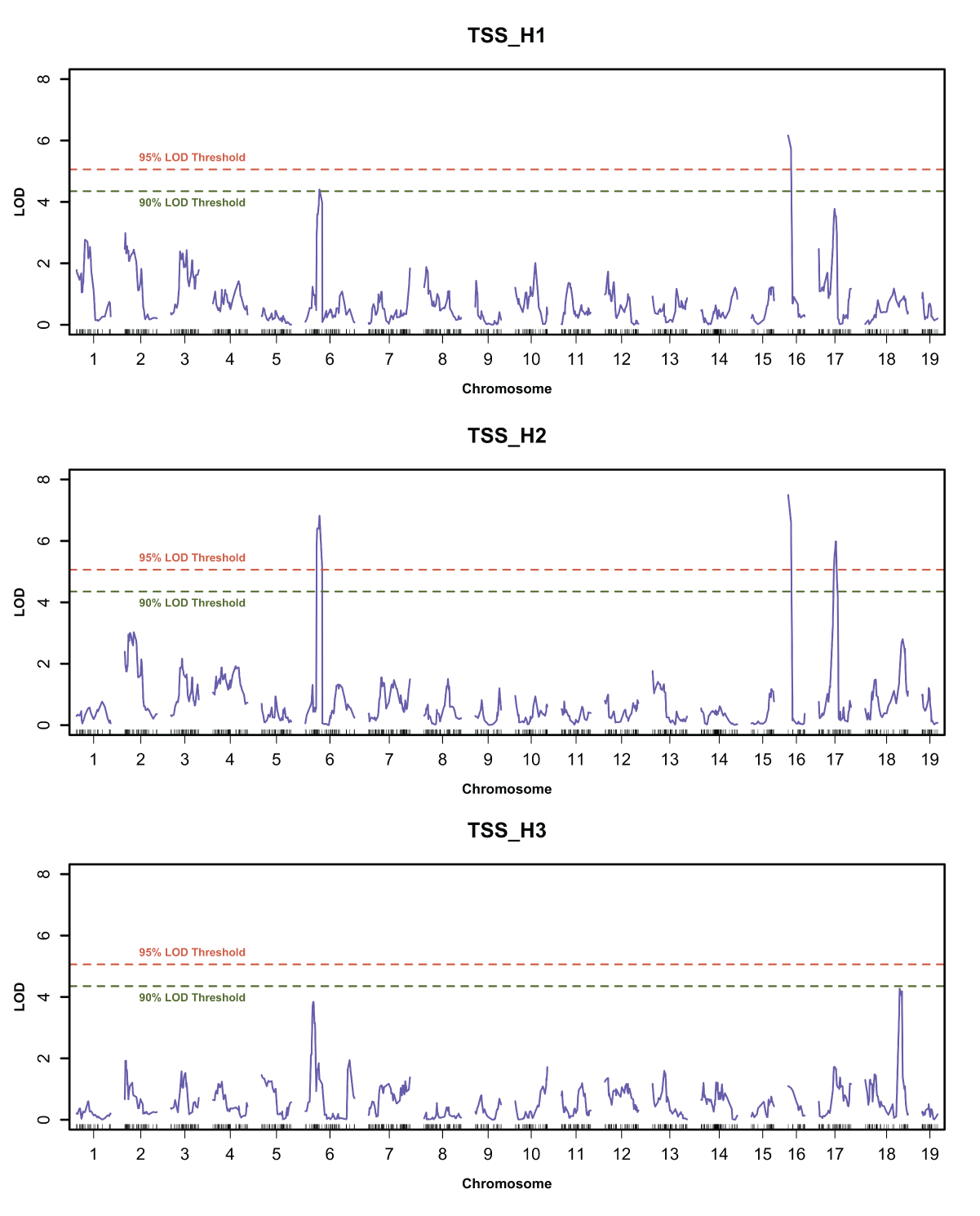

Supplement: Web_Material_uhaf353 [file web_material_uhaf353.zip › sup_fig_S4.png]

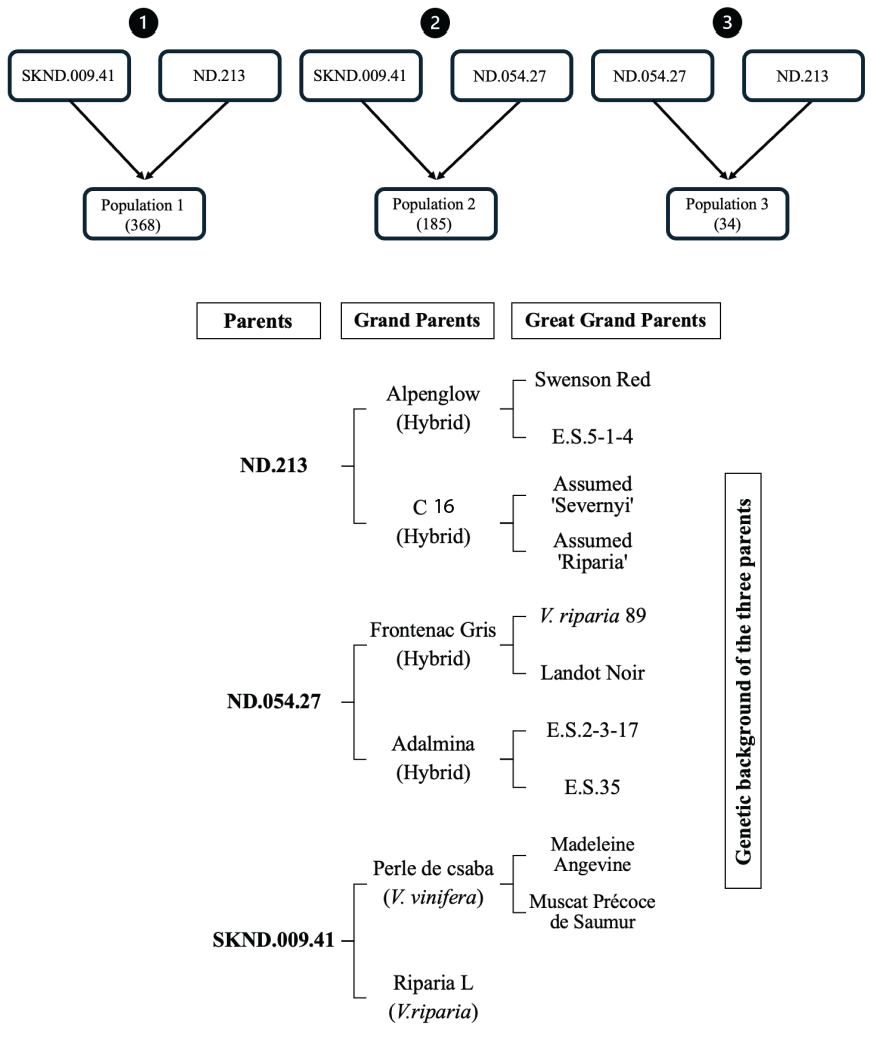

Supplement: Web_Material_uhaf353 [file web_material_uhaf353.zip › Sup_fig_S8.png]
